# Inverse‐Designed On‐Chip Orbital Angular Momentum Mode Converter for Optical Convolution Acceleration

**DOI:** 10.1002/nap2.70002

**Published:** 2026-01-30

**Authors:** Yumeng Chen, Kuo Zhang, Kun Liao, Xiaoyong Hu, Qihuang Gong

**Affiliations:** ^1^ State Key Laboratory for Mesoscopic Physics & Department of Physics, Collaborative Innovation Center of Quantum Matter & Frontiers Science Center for Nano‐optoelectronics Peking University Beijing China; ^2^ State Key Laboratory of Advanced Optical Communication Systems and Networks, Department of Electronic Engineering Shanghai Jiao Tong University Shanghai China; ^3^ Key Laboratory for Advanced Optoelectronic Integrated Chips of Jiangsu Province, Peking University Yangtze Delta Institute of Optoelectronics Nantong Jiangsu China; ^4^ Collaborative Innovation Center of Extreme Optics Shanxi University Taiyuan Shanxi China; ^5^ Hefei National Laboratory Hefei China

**Keywords:** inverse design, optical convolution, optical neural network, orbital angular momentum

## Abstract

Optical neural networks leverage the inherent parallelism of light to multiplex across various degrees of freedom including wavelength, polarization, and modes. Among these, orbital angular momentum (OAM), possessing a theoretically infinite number of orthogonal mode dimensions, holds significant potential for constructing optical neural networks. However, OAM conversion and multiplexing on integrated photonic chips remain challenging. Here, we present an on‐chip OAM mode converter and multiplexer device based on inverse design. The OAM mode converter achieves maximum up‐conversion efficiency of 88.68% (${\text{OAM}}_{-1\to -2}$), maximum down‐conversion efficiency of 88.04% (${\text{OAM}}_{-3\to -1}$), and maximum modulation depth of 4.07 dB (${\text{OAM}}_{+1\to +3}$). Besides, the ${\text{OAM}}_{\pm 1,\pm 2}$ multiplexer achieves maximum conversion efficiency of 98.29% and maximum modulation depth of 20.69 dB. Subsequently, we demonstrate an OAM‐encoded hybrid optical convolutional neural network built using this device, achieving 98.0% accuracy on MNIST handwritten digit recognition and 86.1% accuracy on Fashion‐MNIST classification. This device provides a novel approach for on‐chip OAM conversion and multiplexing while also enabling on‐chip optical convolution operations by using OAM mode. This work offers a practical pathway for integrating OAM with on‐chip optical neural networks.

## Graphical abstract

This work proposes OAM‐encoded CNN based on cascaded on‐chip OAM converters and multiplexers. The footprint of inverse‐designed converters is 5 μm × 4 μm and the maximum conversion efficiency of OAM reaches 88%. The OAM‐encoded optical CNN was built using this device, achieving 98.0% accuracy on MNIST and 86.1% accuracy on Fashion‐MNIST classification. This device offers a practical pathway for integrating OAM with on‐chip optical neural networks.

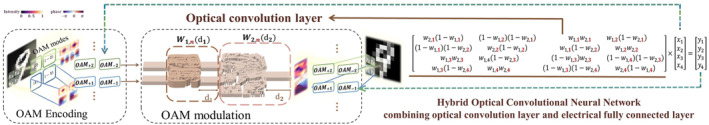

## 1 Introduction

With the exponential growth in computational demands of artificial intelligence (AI), conventional electronic neural networks are confronting insurmountable bottlenecks in power consumption, heat dissipation, and processing speed [1, 2, 3, 4]. Optical neural networks (ONNs) have emerged as a promising solution, leveraging the unique properties of photons to perform computations at the speed of light with potentially lower energy consumption [5, 6]. Unlike electrons, photons enable massively parallel processing through wavelength division multiplexing, exhibit minimal crosstalk, and generate negligible heat during propagation [7]. Recent demonstrations have shown that optical systems can achieve matrix‐vector multiplications—the cornerstone of neural network operations [8, 9]. Furthermore, optical computing naturally supports analog operations, enabling continuous‐valued computations without the quantization errors inherent in digital systems [10, 11, 12, 13, 14].

The true potential of optical computing lies in its ability to exploit multiple degrees of freedom simultaneously. Beyond traditional amplitude and phase modulation, optical systems can harness wavelength, polarization, spatial modes, and orbital angular momentum (OAM) to encode and process information in parallel [15, 16, 17, 18, 19, 20]. This multiplexing capability enables unprecedented information density and computational throughput within compact photonic architectures [21]. Among these dimensions, OAM has attracted particular attention due to its unique mathematical properties and potential for high‐dimensional encoding [22].

Orbital angular momentum represents a particularly attractive multiplexing dimension for optical computing applications. Light beams carrying OAM possess helical phase fronts characterized by an azimuthal phase dependence of exp(ilφ), where l is the topological charge that can theoretically take any integer value [23, 24]. This property enables the creation of an infinite set of orthogonal modes, each distinguishable by its unique topological charge [25]. The orthogonality of OAM modes ensures minimal crosstalk between channels, making them ideal for parallel information processing [26]. Recent experiments have demonstrated the generation and detection of OAM modes with topological charges exceeding |l|=100, highlighting the vast information capacity available through this degree of freedom [27].

Current approaches for OAM generation in optical computing predominantly rely on spatial light modulators (SLMs) and diffractive optical elements. Several works have successfully integrated OAM‐based processing with diffractive neural networks, achieving impressive classification accuracies for image recognition tasks [29, 30]. Metasurface‐based designs have enabled ultracompact OAM multiplexers operating across multiple wavelengths simultaneously [26]. Dynamic holographic techniques using SLMs have demonstrated reconfigurable OAM‐based matrix operations for optical computing applications [27, 28]. Additionally, free‐space optical systems incorporating spiral phase plates have shown promise for implementing convolutional neural network layers using OAM modes [29, 30, 31]. However, these spatial approaches face significant challenges in terms of system integration and miniaturization, as they typically require bulk optical components and precise alignment, limiting their practical deployment in compact photonic processors [31].

To address integration challenges, researchers have developed on‐chip solutions using laser direct‐writing techniques. Three‐dimensional waveguide structures fabricated via femtosecond laser writing have successfully generated and manipulated OAM modes on photonic chips [32, 33, 34, 35, 36, 37]. Silicon photonics platforms have demonstrated integrated OAM emitters using microring resonators with angular gratings [38, 39]. Polymer‐based waveguide circuits written by two‐photon polymerization have enabled complex OAM mode transformations within centimeter‐scale chips [40]. Some groups have achieved OAM mode sorting and detection using integrated photonic circuits [41, 42, 43]. Nevertheless, the inherent limitations of laser direct‐writing technology result in relatively large device footprints, typically spanning several millimeters to centimeters, which prevents the high‐density integration required for practical optical neural networks [44, 45].

The manipulation and multiplexing of orbital angular momentum on highly integrated photonic chips remain exceptionally challenging. Current fabrication techniques struggle to achieve the precise three‐dimensional geometries required for efficient OAM mode conversion within submillimeter footprints [46]. The coupling between OAM modes and conventional waveguide modes introduces additional complexity in chip design [47]. Mode purity and crosstalk become increasingly problematic as device dimensions shrink [48]. These technical barriers have severely limited the application of OAM in practical optical computing systems, where thousands of processing elements must be integrated on a single chip [49].

Here, we propose the OAM‐encoded hybrid convolutional neural network accelerated by optical convolution (hybrid OCNN) based on the cascaded compact on‐chip OAM converter and multiplexer with the method of inverse design. The iterative process of inverse design combines a gradient descent scheme and partial binarization in re‐optimizing. The feature size of converters is 5 μm × 4 μm. The ${\text{OAM}}_{l=\pm 1}$ and ${\text{OAM}}_{l=\pm 2}$ converter achieves maximum up‐conversion efficiency of 88.68%, maximum down‐conversion efficiency of 87.07%, and maximum modulation depth of 3.87 dB. The ${\text{OAM}}_{l=\pm 2}$ and ${\text{OAM}}_{l=\pm 3}$ converter achieves maximum up‐conversion efficiency of 86.56%, maximum down‐conversion efficiency of 80.67%, and maximum modulation depth of 3.72 dB. The ${\text{OAM}}_{l=\pm 1}$ and ${\text{OAM}}_{l=\pm 3}$ converter achieves maximum up‐conversion efficiency of 80.41%, maximum down‐conversion efficiency of 88.04%, and maximum modulation depth of 4.07 dB. The ${\text{OAM}}_{\pm 1,\pm 2}$ multiplexer achieves maximum conversion efficiency of 98.29% and maximum modulation depth of 20.69 dB.

**FIGURE 1:**
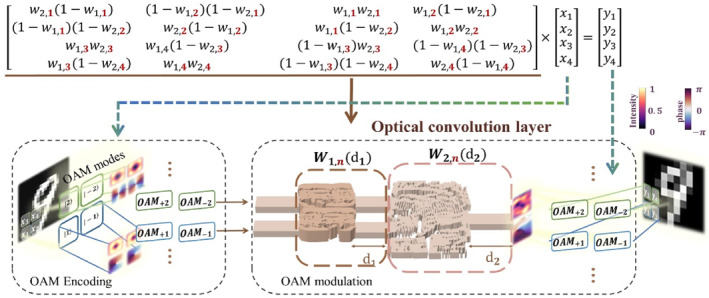
A schematic diagram of the optical convolutional layer in OAM‐encoded hybrid OCNN. Hybrid OCNN utilizes ${\text{OAM}}_{l=\pm 1}$ and ${\text{OAM}}_{l=\pm 2}$ to encode the input data of images and is composed of one optical convolutional layer and one electric fully connected layer. The output data are vectors of which the max values represent the result of classification. The optical convolutional layer in our work is composed of two OAM mode converters and one OAM multiplexer. Convolution operations are achieved by the OAM‐basis transmission matrix of the whole structure which acts as the kernel. We get different kernels to compute the convolution by adjusting the length of output waveguides for the converters and the multiplexers $d_{1}$ and $d_{2}$.

By leveraging the orthogonality of OAM modes, we employ OAM encoding as vector elements to implement convolution operations. The cascaded converter applies weighting to the input OAM modes and the output intensity of OAM modes, which index the result of convolution. Our simulations propose an OAM‐encoded convolutional neural network accelerated by optical convolution (hybrid optical convolutional neural network, combining optical convolution and electrical fully connected layers), as shown in Figure 1. For the task of MINST‐handwritten digit recognition, OAM‐encoded hybrid OCNN achieves an accuracy rate of 98%, compared to 93% for electrical networks with the same amount of training parameters. For the task of Fashion‐MINST classifying 10 distinct objects, OAM‐encoded hybrid OCNN achieved an accuracy rate of 86%, compared to 84% for electrical convolution networks. Our design theoretically provides a novel approach for neural network optical chips demonstrating on OAM modes and proposes a feasible path for OAM applications and integrated optoelectronic computing, as well as the optical acceleration of electrical neural network computing.

## 2 Methods

### 2.1 Inverse Design Method of OAM Mode Converters and Multiplexers

**FIGURE 2:**
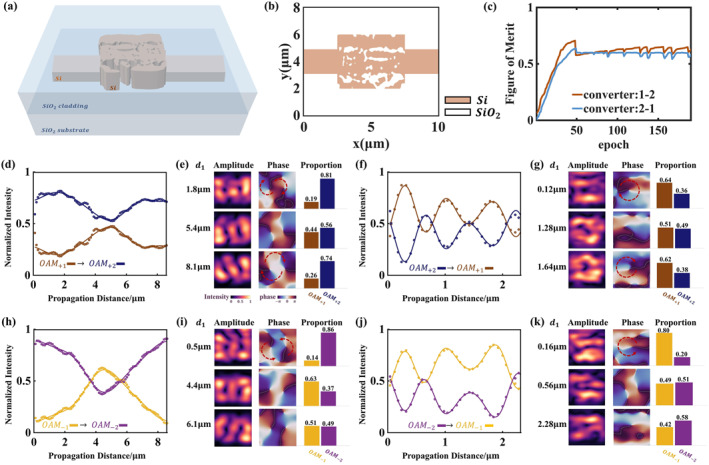
The characterization for the design process, conversion efficiency curves, and the output optical fields of the ${\text{OAM}}_{1↔2}$ converter. (a) A schematic diagram of OAM mode converters. ${\text{OAM}}_{l=\pm n}$ is enabled at the input waveguide and ${\text{OAM}}_{l=\pm m}$ is generated at the output port. (b) Refractive index distributions of the designed converter ${\text{OAM}}_{1↔2}$. (c) Figure of merit the inverse design progress of the ${\text{OAM}}_{1↔2}$ converter. (d) Periodic evolution of the mode fractions for ${\text{OAM}}_{l=+1}$ into ${\text{OAM}}_{l=+2}$ with the output distance. (f) Periodic evolution of the mode fractions for ${\text{OAM}}_{l=+2}$ into ${\text{OAM}}_{l=+1}$ with the output distance. (h) Periodic evolution of the mode fractions for ${\text{OAM}}_{l=-1}$ into ${\text{OAM}}_{l=-2}$ with the output distance. (j) Periodic evolution of the mode fractions for ${\text{OAM}}_{l=-2}$ into ${\text{OAM}}_{l=-1}$ with the output distance. Panels (e, g, i, k) are the intensity, phase, and proportion diagrams of the output light fields at three different positions.

We use the topology optimization schemes which is based on gradient descent to facilitate the inverse design of the OAM mode converters and multiplexers and propose the method of adjusting the optimization weights and partial binarization in re‐optimizing to improve the efficiency and effectiveness of inverse designed devices. The OAM mode converter is designed for fabrication on a silicon (Si) thin film with a thickness of 1800 nm, which is deposited on a silicon dioxide (SiO_2_) substrate with a thickness of 500 μm. The design region is 5 μm × 4 μm × 1800 nm, consisting of 201 × 161 identical material units, each with a size of 25 nm × 25 nm × 1800 nm. The waveguides which can support high order modes at 1550 nm is 1200 nm thick and 1800 nm wide. The OAM multiplexer is designed for fabrication on a 1200‐nm Si thin film deposited on the same SiO_2_ substrate and the design region is 12 μm × 10 μm × 1200 nm, consisting of 481 × 401 identical material units of the same size as the converter. It should be noted that the inverse design region and the waveguide region feature different etch depths to facilitate easier conversion between different OAM orders as illustrated in Figure 2a and the multiplexer keeps the same thickness of the waveguides. Topology optimization process is encoded with Python and MATLAB to process simulation results from the finite‐difference time‐domain method software, Lumerical FDTD Solutions, and calculate the adaptive refractive index.

Firstly, we define the performance of our device as the mode conversion efficiency between the input mode and the output mode at 1550 nm. Target OAM mode is predefined and kept unchanged during the optimization process, detailed in Supporting Information S1: Section S1. We calculate the figure of merit from an overlap integral between the output fields recorded in Lumerical FDTD Solutions and the target OAM mode.

The objective function is defined as

(1) $$\begin{array}{c} f=f\left(E\left(\varepsilon \right)\right)=k_{1}\cdot f_{1}\left(E_{\text{out}}\right)-k_{2}\cdot f_{2}\left(E_{\text{out}}\right), \end{array}$$

where E(ε) is the intensity distribution of the propagation field and $\varepsilon^{k}$ is the permittivity of the *k*th iteration. $f_{1}\left(E_{\text{out}}\right)$ is the ratio of the optical power of the target OAM mode to the incident power of the input OAM mode, calculated via the overlap integral of the simulated output field and the target mode field. $f_{2}\left(E_{\text{out}}\right)$ is the fraction of incident power lost due to propagation, scattering, and converting.

(2) $$\begin{array}{c} f_{1}\left(E_{\text{out}}\right)=\frac{{\left|\iint {E_{\text{out}}}^{*}\left(y,z\right)\cdot E_{\text{target}}\left(y,z\right)ds\right|}^{2}}{\iint {\left|E_{\text{out}}\left(y,z\right)\right|}^{2}ds\iint {\left|E_{\text{target}}\left(y,z\right)\right|}^{2}ds}. \end{array}$$

(3) $$\begin{array}{c} f_{2}\left(E_{\text{out}}\right)=1-\frac{\iint {\left|E_{\text{out}}\left(y,z\right)\right|}^{2}ds}{\iint {\left|E_{\text{in}}\left(y,z\right)\right|}^{2}ds}. \end{array}$$

Here, $E_{\text{in}}\left(y,z\right)$ and $E_{\text{target}}\left(y,z\right)$ are the electric field distributions of the input and target OAM modes, respectively. ds is the cross‐sectional areas of the input and output waveguides. Weight coefficients $k_{1}$ and $k_{2}$ balance performance priorities. In this study, weight coefficients prioritizes conversion efficiency while ensuring low loss and are dynamically adjusted as the different partials of FOM vary.

Secondly, the gradient $\frac{\partial f}{\partial \varepsilon^{k}}$ can be calculated by the equation below:

(4) $$\begin{array}{c} \frac{\partial f}{\partial \varepsilon^{k}}=\frac{\partial E_{\text{out}}}{\partial \varepsilon^{k}}\frac{\partial f}{\partial E_{\text{out}}}. \end{array}$$

Then, we can substitute $\frac{\partial E_{\text{out}}}{\partial \varepsilon^{k}}$ for $k_{1}E_{\text{target}\_\text{mode}}-k_{2}\omega^{2}\mu_{0}E^{\prime }E_{\text{all}\_\text{mode}}$, which is the propagation field intensity distribution of the original source over the designed section, and $\frac{\partial f}{\partial E_{\text{out}}}$ for $E^{\prime }$, which is the propagation field distribution of the adjoint source over the designed region [50, 51, 52].

(5) $$\begin{array}{c} \frac{\partial f}{\partial \varepsilon^{k}}=k_{1}\omega^{2}\mu_{0}E^{\prime }E_{\text{target}\_\text{mode}}-k_{2}\omega^{2}\mu_{0}E^{\prime }E_{\text{all\_mode}}, \end{array}$$

where ω is the frequency of the signal light, and $\mu_{0}$ is the vacuum permeability.

Because of the reciprocity, for the ${\text{OAM}}_{m\to n}$ converter, if we swap the input and output, it can function as a converter for ${\text{OAM}}_{n\to m}$. Both the converting efficiencies of ${\text{OAM}}_{m\to n}$ and ${\text{OAM}}_{n\to m}$ are taken into consideration when we define the FOM of ${\text{OAM}}_{m↔n}$. And both the coupling efficiencies of ${\text{OAM}}_{m}$ and ${\text{OAM}}_{n}$ are taken into consideration when it comes to the multiplexer.

Considering the existence of several input sources, the gradient is written in the form of summary:

(6) $$\begin{array}{c} \varepsilon^{k+1}=\varepsilon^{k}+\sum_{n=1}^{N}\alpha_{n}\frac{\partial f_{n}}{\partial \varepsilon^{k}}. \end{array}$$

Taking into account that the OAM mode converter enables simultaneous conversion of both positive and negative modes, along with up‐conversion and down‐conversion functionalities when the input and output are swapped, the value of *N* is set to 4 in this context. These four conversion scenarios correspond to m→n, −m→−n, n→m, and −n→−m, respectively. $\alpha_{n}$ is used to adjust the intensity differences arising from the light field distribution of different modes and compensate for modes with lower transmittance [38, 39, 40].

(7) $$\alpha_{n}=\frac{\;\frac{\partial f_{n}}{\partial \varepsilon^{k}}}{\min\left(\frac{\partial f_{n}}{\partial {\varepsilon_{i}}^{k}}\right)}\times \left(\sum_{i=1}^{N}T_{i}-T_{n}\right).$$

In the first step of optimization, the refractive index of the material unit in the design region continuously changed between the refractive index of Si ($n_{\text{Si}}=3.48$) and SiO_2_ ($n_{\text{SiO}2}=1.44$), detailed in Supporting Information S1: Section S1.

**FIGURE 3:**
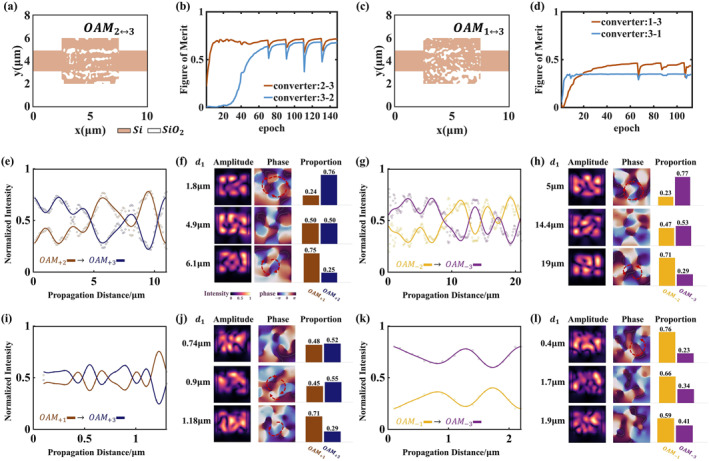
The characterization for the design process, conversion efficiency curves, and the output optical fields of the ${\text{OAM}}_{2↔3}$ converter and the ${\text{OAM}}_{1↔3}$ converter. (a) Refractive index distributions of the designed converters ${\text{OAM}}_{2↔3}$. (b) Figure of merit the inverse design progress of ${\text{OAM}}_{2↔3}$. (c) Refractive index distributions of the designed converters ${\text{OAM}}_{1↔3}$. (d) Figure of merit the inverse design progress of ${\text{OAM}}_{1↔3}$. (e) Periodic evolution of the mode fractions for ${\text{OAM}}_{l=+2}$ into ${\text{OAM}}_{l=+3}$ with the output distance. (g) Periodic evolution of the mode fractions for ${\text{OAM}}_{l=-2}$ into ${\text{OAM}}_{l=-3}$ with the output distance. (i) Periodic evolution of the mode fractions for ${\text{OAM}}_{l=+1}$ into ${\text{OAM}}_{l=+3}$ with the output distance. (k) Periodic evolution of the mode fractions for ${\text{OAM}}_{l=-1}$ into ${\text{OAM}}_{l=-3}$ with the output distance. Panels (f, h, j, l) are the intensity, phase, and proportion diagrams of the output light fields at three different positions.

The second step is partial‐binarization optimization. In this step, the adjoint method is applied, and the refractive index of the material units in the design region is controlled according to the bias factor *β*. We delineate two binarization‐sensitive regions where β varies slowly and other regions where β increases fast to 100. After the completion of binarizing most area, we then binarize these two delineated areas for the additional number of iterations. The convergence in the inverse design progress for the three OAM mode converters is shown in Figures 2c and 3b,d. In the end, $\varepsilon_{i}$ becomes either the value of the refractive index of Si or SiO_2_, as shown in Figures 2b and 3a,c.

## 3 Results

### 3.1 Characterization of OAM Mode Converters

We utilize inverse design to obtain OAM mode converters, which transform ${\text{OAM}}_{l=\pm m}$ to ${\text{OAM}}_{l=\pm n}$, and the conversion efficiency exhibits a periodic variation as the length of the output waveguide increases. In the following section, we will provide a detailed characterization of the functionalities of three converters, transforming between topological Charges 1 and 2, between 2 and 3, and between 1 and 3, respectively.

It should be noted that OAM modes are not intrinsic modes of rectangular waveguides. When an OAM mode propagates in a rectangular waveguide, it essentially consists of a series of intrinsic TE modes of the waveguide propagating together. These superimposed TE modes have different propagation constants, leading to changes in the phase difference between modes during propagation and variations in the field distribution with distance. The OAM field observed in the rectangular waveguide is actually a distorted field of these TE modes, rather than the pure spiral phase and annular intensity distribution of an ideal OAM mode. Within a propagation distance on the order of the wavelength, we inversely treat the TE modes propagating in the waveguide as a superposition of several OAM modes and consider that the propagation distance will affect the content of different OAM modes. As the propagation distance gradually increases, due to modal dispersion, the phase rotation characteristics of this asymmetric light field weaken increasingly, eventually transforming into a stably propagating high‐order TE intrinsic mode. Taking advantage of this characteristic, our compact on‐chip device utilizes rectangular waveguides to connect and adjust the different conversion ratios of the converter for OAM modes.

We characterize the performance of the device using four metrics: conversion efficiency, modulation depth, mode purity, and crosstalk. Among these, conversion efficiency and modulation depth determine the transmission matrix, which represents the modulation range of the weights in the neural network, whereas mode purity and crosstalk influence the accuracy of the results during convolution operations.

When considering the performance of the converter ${\text{OAM}}_{m\to n}$ in encoding and computing, we mainly talk about the output mode to become the OAM mode with the order n and the origin input mode with the order m. The output optical field $f_{\text{out}}$ can be described as follows:

(8) $$\begin{array}{c} f_{\text{out}}=\sum_{l}a_{l}E_{\text{OAM}}\left(r,\varphi ,l\right), \end{array}$$

(9) $$\begin{array}{c} a_{l}=\iint {E_{n}\left(r,\varphi ,l\right)}^{*}\cdot f_{\text{out}}drd\varphi , \end{array}$$

where $E_{n}\left(r,\varphi ,l\right)$ denotes the electric field of the normalized standard OAM mode and $a_{l}$ is the weight coefficient calculated by overlap integral and ${\left|a_{l}\right|}^{2}$ represents the power fraction of the lth order OAM mode in the output optical field and the conversion efficiency is obtained by dividing the energy of ${\text{OAM}}_{n}$ in the output optical field by the sum of these two energies of ${\text{OAM}}_{m}$ and ${\text{OAM}}_{n}$.

(10) $$\begin{array}{c} {C\text{onversion}\;\text{efficiency}}_{m\to n}=\frac{{a_{n}}^{2}}{{a_{n}}^{2}+{a_{m}}^{2}}. \end{array}$$

(11) $$\begin{array}{c} T\text{he}\;\text{modulation}\;\text{depth}=10\;\log\left(\frac{\max\;\text{conversion}\;\text{efficiency}\;}{\min\;\text{conversion}\;\text{efficiency}}\right). \end{array}$$

When accurately evaluating the orthogonality of OAM modes in transmission and communication, mode purity and crosstalk are significance metrics. We define the mode purity of the converter ${\text{OAM}}_{m\to n}$ as the power proportion of ${\text{OAM}}_{n}$ at the output port of the converter ${\text{OAM}}_{m\to n}$ and the crosstalk as the other modes at the output port.

(12) $$\begin{array}{c} {\text{Purity}}_{m\to n}=\frac{{a_{l=n}}^{2}}{\sum_{i=-\infty }^{+\infty }{a_{l=i}}^{2}}. \end{array}$$

(13) $$\begin{array}{c} C\text{rosstalk}=10\;\mathrm{lg}\left(1-\frac{{a_{l=n}}^{2}}{\sum_{i=-\infty }^{+\infty }{a_{l=i}}^{2}}\right). \end{array}$$

Detailed data of conversion efficiency and modulation depth are listed in Tables 1 and 2. Mode purity and crosstalk are provided in the Supporting Information S1: Section S5.

**TABLE 1:** Conversion efficiency and loss of converters.

Converter		Up‐conversion efficiency range	Up‐modulation depth (dB)	Down‐conversion efficiency range	Down‐modulation depth (dB)	Average loss (dB)
${\text{OAM}}_{1↔2}$	+1↔+2	52.81%to81.54%	1.89	36.91%to87.07%	3.73	−2.24
	−1↔−2	37.22%to88.68%	3.87	42.36%to83.66%	2.96	
${\text{OAM}}_{2↔3}$	+2↔+3	36.73%to86.56%	3.72	36.07%to80.67%	3.50	−2.40
	−2↔−3	45.08%to80.31%	2.51	44.60%to79.87%	2.53	
${\text{OAM}}_{1↔3}$	+1↔+3	24.52%to62.56%	4.07	36.78%to65.36%	2.50	−2.64
	−1↔−3	59.86%to80.41%	1.28	49.39%to88.04%	2.51	

**TABLE 2:** Conversion efficiency and modulation depth of ${\text{OA}M}_{\pm 1,\pm 2}$ multiplexer.

${\text{OAM}}_{\pm 1,\pm 2}$ multiplexer	Conversion efficiency range	Modulation depth (dB)
+1↔−1	1.99%to97.40%	16.90
−1↔+1	0.83%to97.25%	20.69
+2↔−2	9.77%to98.29%	10.03
−2↔+2	9.65%to98.17%	10.07

#### 3.1.1 ${\text{OAM}}_{1↔2}$ Converter

The converter can transform ${\text{OAM}}_{l=+1}$ into ${\text{OAM}}_{l=+2}$ and ${\text{OAM}}_{l=-1}$ mode into ${\text{OAM}}_{l=-2}$. Conversely, it can also transform ${\text{OAM}}_{l=+2}$ into ${\text{OAM}}_{l=+1}$ and ${\text{OAM}}_{l=-2}$ into ${\text{OAM}}_{l=-1}$. We fit the variation curves of the content ratios of the second‐order and first‐order modes in the output as a function of the output waveguide length, as shown in Figure 2d,f,h,j. Since the proportions of the output second‐order and first‐order modes vary periodically with the length of the output waveguide as shown in Figure 2e,g,i,k, we employed trigonometric functions to fit the curves. Due to the orthogonality between different orders of OAM modes, the four superimposed OAM modes can be regarded as four basis vectors |m〉, |−m〉, |n〉, |−n〉(m=1,n=2). The conversion between the two modes is expressed as

(14a) $$\begin{array}{c} |+m\rangle \to \left(1-\alpha_{mn}\right)|+m\rangle +\alpha_{mn}|+n\rangle . \end{array}$$

(14b) $$\begin{array}{c} |-m\rangle \to \left(1-\alpha_{-m-n}\right)|-m\rangle +\alpha_{-m-n}|-n\rangle . \end{array}$$

(14c) $$\begin{array}{c} |+n\rangle \to \alpha_{nm}|+m\rangle +\left(1-\alpha_{nm}\right)|+n\rangle . \end{array}$$

(14d) $$\begin{array}{c} |-n\rangle \to \alpha_{-n-m}|-m\rangle +\left(1-\alpha_{-n-m}\right)|-n\rangle . \end{array}$$

Here, $\alpha_{mn}$ denotes the conversion efficiency from ${\text{OAM}}_{l=m}$ to ${\text{OAM}}_{l=n}$ (m,n=1,−1,2,−2). The input superimposed OAM modes can be expressed as $\left[x_{1},x_{2},x_{3},x_{4}\right]$. The overall transmission matrix of the on‐chip converter device can be written as

(15) $$\begin{array}{c} w_{mn}=\left[\begin{array}{cccc} 1-\alpha_{mn} & 0 & \alpha_{mn} & 0\\ 0 & 1-\alpha_{-m-n} & 0 & \alpha_{-m-n}\\ \alpha_{mn} & 0 & 1-\alpha_{nm} & 0\\ 0 & \alpha_{-n-m} & 0 & 1-\alpha_{-n-m} \end{array}\right]. \end{array}$$

The detailed parameters of the curve fit are listed in Supporting Information S1: Section S2.

#### 3.1.2 ${\text{OAM}}_{2↔3}$ Converter and ${\text{OAM}}_{1↔3}$ Converter

For the ${\text{OAM}}_{2↔3}$ converter and ${\text{OAM}}_{1↔3}$ converter, we also fit the variation curves of the ratios as shown in Figure 3e,g,i,k and express the input superimposed OAM modes’ four basis vectors |m〉, |−m〉, |n〉, |−n〉 as $\left[x_{1},x_{2},x_{3},x_{4}\right]$ as shown in Figure 3f,h,j,l. For the ${\text{OAM}}_{2↔3}$ converter, m=2, n=3. For the ${\text{OAM}}_{1↔3}$ converter, m=1, n=3. The detailed parameters of the curve fit for the transmission matrix are listed in Supporting Information S1: Section S2.

The conversion efficiency, modulation depth, and average loss of three converters are shown in Table 1.

#### 3.1.3 Multiplexer for ${\text{OAM}}_{\pm 1}$ and ${\text{OAM}}_{\pm 2}$

**FIGURE 4:**
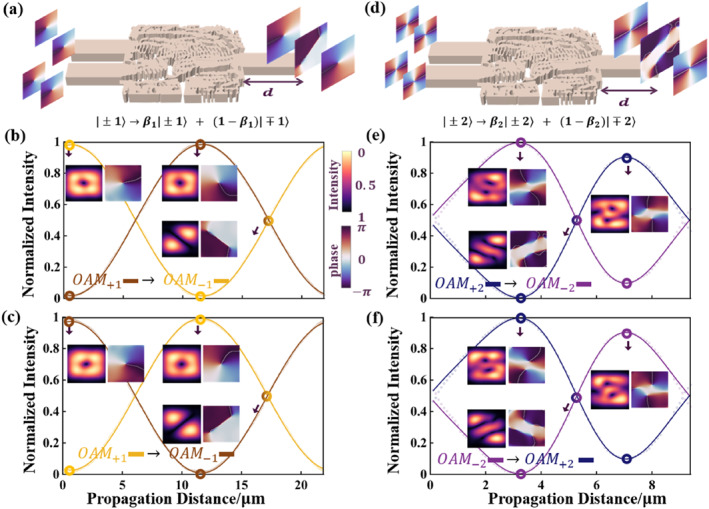
The conversion efficiency curves and the output optical fields of the OAM multiplexer. A schematic diagram of the OAM multiplexer of (a) ${\text{OAM}}_{l=\pm 1}$ (d) ${\text{OAM}}_{l=\pm 2}$. Periodic evolution of the mode fractions with the output distance and the intensity, phase, and proportion diagrams of the output light fields at three different positions when the input field is (b) ${\text{OAM}}_{l=+1}$, (c) ${\text{OAM}}_{l=-1}$, (e) ${\text{OAM}}_{l=+2}$, and (f) ${\text{OAM}}_{l=-2}$.

${\text{OAM}}_{l=\pm 1,\pm 2}$ are enabled at the input ports and the superposition OAM modes after proportion adjustment are emitted from the output port as shown in Figure 4a,d. The multiplexer can achieve transformation between ${\text{OAM}}_{l=+m}$ and ${\text{OAM}}_{l=-m}$. We also fit the variation curves of the ratios and the four basis are still |m〉, |−m〉, |n〉, |−n〉(m=1,n=2) consisting the input vectors $\left[x_{1},x_{2},x_{3},x_{4}\right]$, as shown in Figure 4b,c,e,f. The coupling effect between the two modes is expressed as

(16a) $$\begin{array}{c} |\pm m\rangle \to \beta_{m}|\pm m\rangle +\left(1-\beta_{m}\right)|\mp m\rangle . \end{array}$$

(16b) $$\begin{array}{c} |\pm n\rangle \to \beta_{n}|\pm n\rangle +\left(1-\beta_{n}\right)|\mp n\rangle . \end{array}$$

The overall transmission matrix of the multiplexer can be written as

(17) $$w_{2}=\left[\begin{array}{cccc} \beta_{m} & 1-\beta_{m} & 0 & 0\\ 1-\beta_{m} & \beta_{m} & 0 & 0\\ 0 & 0 & \beta_{n} & 1-\beta_{n}\\ 0 & 0 & 1-\beta_{n} & \beta_{n} \end{array}\right].$$

Here, $\beta_{m}$ denotes the conversion efficiency from ${\text{OAM}}_{l=m}$ to ${\text{OAM}}_{l=-m}$ (m=1,−1), and $\beta_{n}$ denotes the conversion efficiency from ${\text{OAM}}_{l=n}$ to ${\text{OAM}}_{l=-n}$ (n=2,−2). The detailed parameters of the curve fit are listed in Supporting Information S1: Section S2.

The conversion efficiency and modulation depth of ${\text{OAM}}_{\pm 1,\pm 2}$ multiplexer are shown in Table 2.

### 3.2 OAM Mode Converters for Hybrid OCNN Acceleration

**FIGURE 5:**
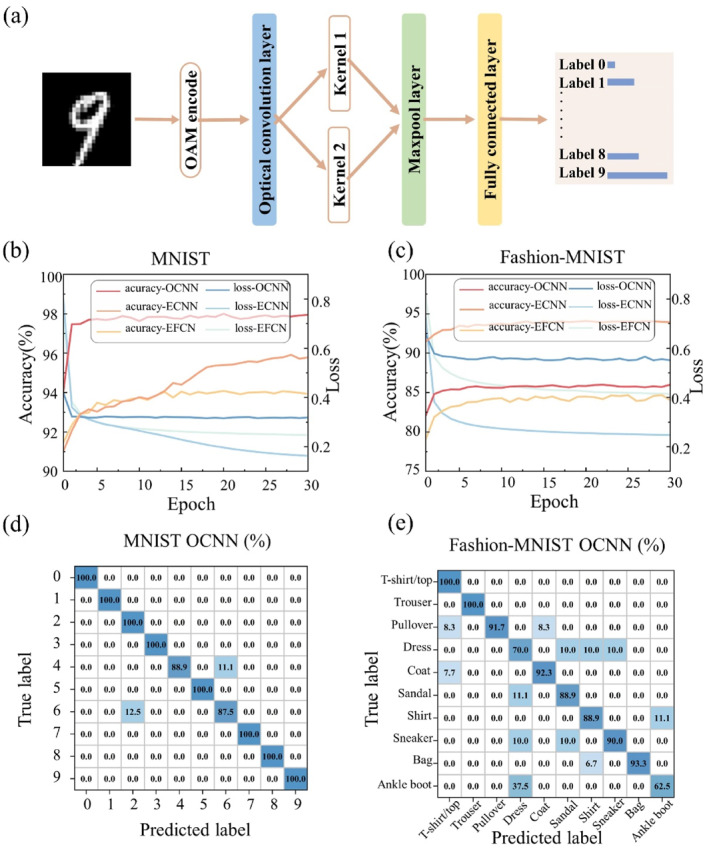
Systemic construction and evaluation of OAM‐encoded hybrid OCNN. (a) A schematic workflow of OAM‐encoded hybrid OCNN. (b) Convergence of the hybrid OCNN, ECNN, and EFCN for the MNIST handwritten digit dataset. (c) The confusion matrix of the hybrid OCNN, ECNN, and EFCN in the MNIST handwritten digit dataset. (d) The confusion matrix of the hybrid OCNN in the MNIST handwritten digit dataset. (e) The confusion matrix of the hybrid OCNN in the Fashion‐MNIST clothing item dataset.

We further employ the designed ${\text{OAM}}_{1↔2}$ converters as convolution kernel acceleration to accelerate the convolution operation in image processing. Two symmetric OAM mode converters (for up‐conversion from 1 to 2 and down‐conversion from 2 to 1, respectively) are placed in parallel and the converted OAM light is emitted from the same output port after passing through a symmetric directional multiplexer, as shown in Figure 5a.

First, the image data are normalized to the range of 0∼1. The data of four pixel points with coordinates (m,n), (m,n+1), (m+1,n), and (m+1,n+1), $\left({x_{1}}^{2},{x_{2}}^{2},{x_{3}}^{2},{x_{4}}^{2}\right)$ are encoded into the light intensities of OAM with orders of +1, −1, +2, and −2, respectively, denoted as the input:

(18) $$\begin{array}{c} x={\;\left[x_{1},x_{2},x_{3},x_{4}\right]}^{T}. \end{array}$$

The two input light beams of the up waveguide and the down waveguide carrying different OAM modes are

(19) $$\begin{array}{ll} f_{\text{input}} & =f_{\text{up}\;\text{waveguide}}+f_{\text{down}\;\text{waveguide}}\\ & =\left(x_{1}E_{\text{OAM}}\left(A,\varphi ,l=+1\right)+x_{2}E_{\text{OAM}}\left(A,\varphi ,l=-1\right)\right).\\ & +\left(x_{3}E_{\text{OAM}}\left(A,\varphi ,l=+2\right)+x_{4}E_{\text{OAM}}\left(A,\varphi ,l=-2\right)\right) \end{array}$$

$f_{\text{up}\;\text{waveguide}}$ represents the input light field of the upper waveguide for the ${\text{OAM}}_{1\to 2}$ converter, which has the ${\text{OAM}}_{l=+1}$ and ${\text{OAM}}_{l=-1}$ to encode the two pixels (m,n) and (m,n+1). Similarly, $f_{\text{down}\;\text{waveguide}}$ represents the input light field of the upper waveguide for the ${\text{OAM}}_{2\to 1}$ converter, which has the ${\text{OAM}}_{l=+2}$ and ${\text{OAM}}_{l=-2}$ to encode the two pixels (m+1,n) and (m+1,n+1).

Subsequently, the four input OAM modes pass through the OAM mode converter to obtain

(20) $$x^{\prime }={\;w_{1}\times x=w_{1}\times \left[x_{1},x_{2},x_{3},x_{4}\right]}^{T}=\left[\begin{array}{cccc} 1-w_{1,1} & 0 & w_{1,1} & 0\\ 0 & 1-w_{1,2} & 0 & w_{1,2}\\ w_{1,3} & 0 & 1-w_{1,3} & 0\\ 0 & w_{1,4} & 0 & 1-w_{1,4} \end{array}\right]\times \left[\begin{array}{c} \begin{array}{c} x_{1}\\ x_{2} \end{array}\\ \begin{array}{c} x_{3}\\ x_{4} \end{array} \end{array}\right]=\left[\begin{array}{c} \begin{array}{c} {x_{1}}^{\prime }\\ {x_{2}}^{\prime } \end{array}\\ \begin{array}{c} {x_{3}}^{\prime }\\ {x_{4}}^{\prime } \end{array} \end{array}\right].$$

After passing through the multiplexer, the result is

(21) $$y=w_{2}\times x^{\prime }=\left[\begin{array}{cccc} w_{2,1} & 1-w_{2,1} & 0 & 0\\ 1-w_{2,2} & w_{2,2} & 0 & 0\\ 0 & 0 & w_{2,3} & 1-w_{2,3}\\ 0 & 0 & 1-w_{2,4} & w_{2,4} \end{array}\right]\times \left[\begin{array}{c} \begin{array}{c} {x_{1}}^{\prime }\\ {x_{2}}^{\prime } \end{array}\\ \begin{array}{c} {x_{3}}^{\prime }\\ {x_{4}}^{\prime } \end{array} \end{array}\right]=\left[\begin{array}{c} \begin{array}{c} y_{1}\\ y_{2} \end{array}\\ \begin{array}{c} y_{3}\\ y_{4} \end{array} \end{array}\right].$$

(22) $$\left[\begin{array}{c} \begin{array}{c} y_{1}\\ y_{2} \end{array}\\ \begin{array}{c} y_{3}\\ y_{4} \end{array} \end{array}\right]=\left[\begin{array}{cccc} w_{2,1} & 1-w_{2,1} & 0 & 0\\ 1-w_{2,2} & w_{2,2} & 0 & 0\\ 0 & 0 & w_{2,3} & 1-w_{2,3}\\ 0 & 0 & 1-w_{2,4} & w_{2,4} \end{array}\right]\times \left[\begin{array}{cccc} 1-w_{1,1} & 0 & w_{1,1} & 0\\ 0 & 1-w_{1,2} & 0 & w_{1,2}\\ w_{1,3} & 0 & 1-w_{1,3} & 0\\ 0 & w_{1,4} & 0 & 1-w_{1,4} \end{array}\right]\times \left[\begin{array}{c} \begin{array}{c} x_{1}\\ x_{2} \end{array}\\ \begin{array}{c} x_{3}\\ x_{4} \end{array} \end{array}\right]=\left[\begin{array}{cccc} w_{2,1}\left(1-w_{1,1}\right) & \left(1-w_{1,2}\right)\left(1-w_{2,1}\right) & w_{1,1}w_{2,1} & w_{1,2}\left(1-w_{2,1}\right)\\ \left(1-w_{1,1}\right)\left(1-w_{2,2}\right) & w_{2,2}\left(1-w_{1,2}\right) & w_{1,1}\left(1-w_{2,2}\right) & w_{1,2}w_{2,2}\\ w_{1,3}w_{2,3} & w_{1,4}\left(1-w_{2,3}\right) & \left(1-w_{1,3}\right)w_{2,3} & \left(1-w_{1,4}\right)\left(1-w_{2,3}\right)\\ w_{1,3}\left(1-w_{2,4}\right) & {w_{1,4}w}_{2,4} & \left(1-w_{1,3}\right)\left(1-w_{2,4}\right) & w_{2,4}\left(1-w_{1,4}\right) \end{array}\right]\times \left[\begin{array}{c} \begin{array}{c} x_{1}\\ x_{2} \end{array}\\ \begin{array}{c} x_{3}\\ x_{4} \end{array} \end{array}\right].$$

The output of the ${\text{OAM}}_{1\to 2}$ converter is the superposition of ${\text{OAM}}_{l=+1}$ and ${\text{OAM}}_{l=+2}$ converted from the original ${\text{OAM}}_{l=+1}$, as well as the superposition of ${\text{OAM}}_{l=-1}$ and ${\text{OAM}}_{l=-2}$ converted from the original ${\text{OAM}}_{l=-1}$. And the output of the ${\text{OAM}}_{2\to 1}$ converter is the superposition of ${\text{OAM}}_{l=+1}$ and ${\text{OAM}}_{l=+2}$ converted from the original ${\text{OAM}}_{l=+2}$, as well as the superposition of ${\text{OAM}}_{l=-1}$ and ${\text{OAM}}_{l=-2}$ converted from the original ${\text{OAM}}_{l=-2}$. The output waveguides of the upper ${\text{OAM}}_{1\to 2}$ converter and the lower ${\text{OAM}}_{2\to 1}$ converter serve as the input waveguides of the following multiplexer. During the multiplexing process, ${\text{OAM}}_{l=+1}$ and ${\text{OAM}}_{l=-1}$ undergo mutual conversion, while ${\text{OAM}}_{l=+2}$ and ${\text{OAM}}_{l=-2}$ also undergo mutual conversion. These converted modes are then coupled into a single output waveguide. The multiplexed light beam is expressed as

(23) $$\begin{array}{cc} f_{\text{output}} & =\left(y_{1}E_{\text{OAM}}\left(A,\varphi ,l=+1\right)+y_{2}E_{\text{OAM}}\left(A,\varphi ,l=-1\right)\right)\\ & +\left(y_{3}E_{\text{OAM}}\left(A,\varphi ,l=+2\right)+y_{4}E_{\text{OAM}}\left(A,\varphi ,l=-2\right)\right) \end{array}.$$

The detection and demultiplexing of OAM are based on the principle of spectral decomposition by performing overlap integrals between the output modes and the standard OAM modes. This process can be achieved through the projection measurement between the output modes and the normalized standard OAM mode. A detailed discussion of viable approaches for experimental detection is provided in Supporting Information S1: Section S4. The output is also decomposed into the same four modes as the input.

(24) $$\begin{array}{c} y={\;\left[y_{1},y_{2},y_{3},y_{4}\right]}^{T}. \end{array}$$

The four output modes are stored by the computer and then $\left[{y_{1}}^{2},{y_{2}}^{2},{y_{3}}^{2},{y_{4}}^{2}\right]$ is fed into the subsequent fully connected layer for the following operation in a computer. Afterward, the convolution kernel slides to compute the next position.

Utilizing optical convolution kernels, we construct OAM‐encoded hybrid OCNN and demonstrated its performance on the following two image classification tasks. Below, we will introduce the architecture of the OAM‐encoded hybrid OCNN. Firstly, the input consists of single‐channel grayscale images, such as those from the MNIST handwritten digit dataset and the Fashion‐MNIST clothing item dataset. The size of the input feature map is 24 × 24 (height = 24, width = 24). Secondly, we extract the spatial local features of the input data using an optical convolutional layer. This layer comprises 2 independent convolutional kernels, each of which is of the size of 2 × 2 (height = 2, width = 2) and is with the stride of 2. The two convolutional kernels operate on different positions of the input feature map and respectively they form two output channels. Through this operation, an output feature map with 2 channels is obtained, corresponding to the 24 × 24 output data of the convolutional layer. Subsequently, the 24 × 24 × 2 output data from the convolutional layer are transmitted to the max‐pooling layer, where a 2 × 2 kernel is employed for max‐pooling operation, resulting in a 12 × 12 × 2 output. These outputs are flattened into a 288 × 1 one‐dimensional vector, which is then fed into the subsequent fully connected layer. The fully connected layer generates 10 output values, with a weight matrix of size 288 × 10. The label corresponding to the maximum value among the 10 output values is taken as the final classification result of the input image. The confusion matrixes of the OAM‐encoded hybrid OCNN in the MNIST dataset and in the Fashion‐MNIST dataset are shown in Figure 5d,e.

We tested a traditional electronic neural network on the same dataset and compared the training processes as well as the predictive performance of the two networks as shown in Figure 5b,c. OAM‐encoded hybrid OCNN demonstrates a noticeable improvement in prediction accuracy compared to the traditional fully connected electronic neural network with an equivalent parameter amount. In the task of recognizing handwritten digits from the MNIST dataset, OAM‐encoded hybrid OCNN achieves an accuracy of 98%. This represents a substantial performance advantage of 6 percentage points over the electronic fully connected neural network with an identical parameter count, which only yields an accuracy of 92%. Similarly, on the Fashion‐MNIST dataset, the hybrid OCNN demonstrates superior recognition capability, attaining an accuracy of 86%. The OAM‐encoded hybrid OCNN achieves improvement in accuracy for the image classification task.

## 4 Conclusion

In summary, this study successfully demonstrates multiple on‐chip OAM mode converters via inverse design, enabling efficient conversion between OAM modes with different topological charges. The footprint of the converters is on the order of tens of square micrometers while maintaining high conversion performance, with conversion efficiency reaching 90%. By leveraging the parallel computing advantages of OAM modes, on the MNIST handwritten digit recognition task, the OAM‐encoded hybrid OCNN reached 98% accuracy. On the Fashion‐MNIST dataset, hybrid OCNN attained 86% accuracy. These results indicate that the OAM‐encoded hybrid OCNN can extract image features more effectively, leading to higher accuracy and better performance in image classification tasks.

Although several challenges remain in practical implementation, including the need for redesigned device architectures to multiplex higher‐order OAM modes and potential impacts of fabrication tolerances on inference accuracy, these obstacles present surmountable engineering trade‐offs rather than fundamental limitations. Device dimensions can be moderately increased to accommodate fabrication tolerances while maintaining microscale footprints, orders of magnitude smaller than existing millimeter or centimeter‐scale alternatives. Similarly, higher‐order OAM manipulation can be achieved through more sophisticated topological structures without compromising the essential compactness of the integrated device.

This work establishes a viable pathway for integrating on‐chip OAM manipulation with optical convolutional networks. By encoding OAM modes as neural network processing elements, we successfully demonstrated image classification with competitive accuracy. In subsequent research, device performance can be enhanced by exploring optimized inverse design approaches. Additionally, studying multiplexing techniques based on additional photonic degrees of freedom—such as wavelength division multiplexing—can address classification challenges on more demanding datasets like CIFAR and ImageNet. Therefore, the compact OAM mode converters and multiplexer based on inverse design offers a scalable solution for on‐chip optical convolutional networks, promising significant advancement for multidimensional multiplexing in on‐chip optical convolutional networks.

## Author Contributions

**Yumeng Chen:** conceptualization, investigation, writing – original draft, methodology, validation, visualization, writing – review and editing, software, formal analysis, project administration, data curation, supervision, funding acquisition, resources. **Kuo Zhang:** conceptualization, writing – original draft, methodology, validation, visualization, writing – review and editing, software, formal analysis, project administration, data curation, supervision, funding acquisition, resources. **Kun Liao:** conceptualization, methodology, visualization, writing – review and editing, software, formal analysis, project administration, data curation, supervision, funding acquisition, resources. **Xiaoyong Hu:** conceptualization, funding acquisition, writing – review and editing, formal analysis, project administration, supervision, resources. **Qihuang Gong:** conceptualization, writing – review and editing, project administration, supervision, funding acquisition, resources.

## Conflicts of Interest

The authors declare no conflicts of interest.

## Supporting information


Supporting Information S1

## Data Availability

All data needed to evaluate the conclusions are present in the manuscript and Supporting Information S1.
