# Supplementary material for: Inverse‐Designed On‐Chip Orbital Angular Momentum Mode Converter for Optical Convolution Acceleration
Source: Nanophotonics. 2026 Jan 30;15(3):e70002. doi: 10.1002/nap2.70002 (PMC12965031; doi:10.1002/nap2.70002)
Supplement: Supplementary file 1 — Supporting Information S1 [file NAP2-15-e70002-s001.docx]

**Supplementary materials for Inverse-Designed On-Chip OAM Mode Converter for Optical Convolution Acceleration**

Yumeng Chen, Kuo Zhang, Kun Liao*, Xiaoyong Hu* and Qihuang Gong

**Contents**

**S1. Details for inverse design**

**S2. The curve fit for the** $\mathbf{OAM}_{\boldsymbol{m\leftrightarrow n}}$ **converter and multiplexer**

**S3. Performance comparison among different neural network schemes**

**S4. Feasibility Analysis of OAM Detection and Demultiplexing**

**S5. Power spectrums of three converters and themultiplexe**r

**S6.** **Robustness Analysis Under Crosstalk**

**S7. Theoretical analysis of on-chip OAM order generation**

**S1.** **Details for inverse design**

First of all, the objective function is defined as

$$\begin{aligned} f=f\left( E\left( \varepsilon\right) \right)=k_{1}\cdot f_{1}\left( E_{out} \right)-k_{2}\cdot f_{2}\left( E_{out} \right) \\ =k_{1}\cdot\frac{\left| \iint{E_{out}}^{*}\left( y,z \right)\cdot E_{target}\left( y,z \right)ds \right|^{2}}{\iint\left| E_{out}\left( y,z \right) \right|^{2}ds\iint\left| E_{target}\left( y,z \right) \right|^{2}ds}-k_{2}\cdot(1-\frac{\iint\left| E_{out}\left( y,z \right) \right|^{2}ds}{\iint\left| E_{in}\left( y,z \right) \right|^{2}ds})\#\left( S1 \right) \end{aligned}$$

$f_{1}\left( E_{out} \right)$ is the ratio of the optical power of the target OAM mode to the incident power of the input OAM mode, calculated via the overlap integral of the simulated output field and the target mode field.$f_{2}\left( E_{out} \right)$ is the fraction of incident power lost due to propagation, scattering, and converting [1]. $E_{in}(y,z)$and $E_{target}(y,z)$ are the electric field distributions of the input and target OAM modes, respectively. $ds$ is the cross-sectional areas of the input and output waveguides. Weight coefficients $k_{1}$,$k_{2}$ balances performance priorities. In this study, Weight Coefficients prioritizes conversion efficiency while ensuring low loss and are dynamically adjusted as the different partials of FOM varies.

When changing the relative permittivity $\varepsilon(x,y)$ of a unit at $\left( x,y \right)$ in the design region, we introduce a variation of the electric dipole moment, leading to a variation of the output electromagnetic field simultaneously. Target output modes are pre-defined and keep unchanged during the optimization process. Besides, $H_{output}\propto E_{output}$, so the objective function can be expressed as

$$\begin{aligned} f=f\left( E_{output}\left( \varepsilon\right) \right)\#\left( S2 \right) \end{aligned}$$

where $\varepsilon$is the permittivity distribution of the design region. The total change of the output field will be a superposition of variations caused by every unit in design region.

We use adjoint method to maximize the conversion efficiency and transmission of the target light field and $\varepsilon^{k}$is the permittivity of the $k th$ iteration

$$\begin{aligned} \varepsilon^{k+1}=\varepsilon^{k}+\alpha\frac{\partial f}{\partial\varepsilon^{k}}\#\left( S3 \right) \end{aligned}$$

Secondly, the gradient $\frac{\partial f}{\partial\varepsilon^{k}}$ is calculated by the equation below:

$$\begin{aligned} \frac{\partial f}{\partial\varepsilon^{k}}=\frac{\partial E}{\partial\varepsilon^{k}}\frac{\partial f}{\partial E}\#\left( S4 \right) \end{aligned}$$

From Maxwell's equations, we can get

$$\begin{aligned} \nabla\times\left( \nabla\times E \right)-\omega^{2}\varepsilon\mu_{0}E=i\omega\mu_{0}J\#\left( S5 \right) \end{aligned}$$

where$\omega$ is the frequency of the signal light, $\mu_{0}$ is the vacuum permeability. Eq.(S5) can be written as

$$\begin{aligned} AE=i\omega\mu_{0}J\#\left( S6 \right) \end{aligned}$$

where $A$ represents the operator $\nabla\times\left( \nabla\times\right)-\omega^{2}\varepsilon\mu_{0}$.

Calculating the partial derivative of both sides of Eq.(S6), we get the following equations,

$$\begin{aligned} \frac{\partial A}{\partial\varepsilon^{k}}E+A\frac{\partial E}{\partial\varepsilon^{k}}=0\#\left( S7 \right) \end{aligned}$$

$$\begin{aligned} \frac{\partial E}{\partial\varepsilon^{k}}=A^{-1}\frac{\partial A}{\partial\varepsilon^{k}}E=A^{-1}\cdot\left( -\omega^{2}\mu_{0} \right)\cdot E\#\left( S8 \right) \end{aligned}$$

Then we can use Eq.(S8) to substitute $\frac{\partial E}{\partial\varepsilon^{k}}$ in Eq.(S4) for $E$, which is the propagation field distribution of the original source over the designed region [2,3].

$$\begin{aligned} \frac{\partial f}{\partial\varepsilon^{k}}=-\omega^{2}\mu_{0}A^{-1}\frac{\partial f}{\partial E}E\#\left( S9 \right) \end{aligned}$$

We assume that there is another source $E'$ satisfying the following equation to substitute $\frac{\partial f}{\partial E}$

$$\begin{aligned} AE^{'}=i\omega\mu_{0}J^{'}=\frac{\partial f}{\partial E}\#\left( S10 \right) \end{aligned}$$

Then Eq.(S9) can be written as

$$\begin{aligned} \frac{\partial f}{\partial\varepsilon^{k}}=-\omega^{2}\mu_{0}E^{'}E\#\left( S11 \right) \end{aligned}$$

We consider $E'$ as the propagation field distribution of adjoint light source over the designed section. Then the variation in the objective function $f$ as the variation of $\varepsilon$ can be expressed as

$$\begin{aligned} \frac{\partial f}{\partial{\varepsilon\left( x,y \right)}^{k}}=-\omega^{2}\mu_{0}E^{'}\left( x,y \right)E\left( x,y \right)\#\left( S12 \right) \end{aligned}$$

Thirdly, we perform two simulations, including one forward simulation with the original source input and the adjoint simulation with the adjoint source input [4]. As the two field distributions $E$,$E'$ have been collected and then we can get the gradient of the objective function for the permittivity.

Considering several input sources the gradient is written in the form of summary:

$$\begin{aligned} \varepsilon^{k+1}=\varepsilon^{k}+\sum_{n=1}^{N} \alpha_{n}\frac{\partial f_{n}}{\partial\varepsilon^{k}}\#\left( S13 \right) \end{aligned}$$

as the objective function $f$ is written:

$$\begin{aligned} f=f_{+m}\left( E_{1}\left( \varepsilon\right) \right)+f_{-m}\left( E_{2}\left( \varepsilon\right) \right)+f_{+n}\left( E_{3}\left( \varepsilon\right) \right)+f_{-n}\left( E_{N}\left( \varepsilon\right) \right)\#\left( S14 \right) \end{aligned}$$

$n$ represents the number of input sources. In each iteration, we use 4 OAM modes as input fields respectively and conduct four finite difference time domain method simulations to obtain 4 corresponding gradients, which are then multiply by $\alpha_{n}$and summed up.

Take the $\mathrm{OAM}_{1\leftrightarrow2}$ Converter as an example, we only enable source $\mathrm{OAM}_{+1}$ to get the propagation field distribution$E$ and then we only enable its adjoint source which is also ${OAM'}_{+2}$ at output port to get the propagation field distribution$E'$ so we can calculate $\frac{\partial f_{1}}{\partial\varepsilon^{k}}$. Next, we only enable source $\mathrm{OAM}_{-1}$ and then we only enable its adjoint source which is also ${OAM'}_{-2}$ at output port so we can calculate $\frac{\partial f_{2}}{\partial\varepsilon^{k}}$. Similarly, we can also get $\frac{\partial f_{3}}{\partial\varepsilon^{k}}$ for $\mathrm{OAM}_{+2}$ and $\frac{\partial f_{4}}{\partial\varepsilon^{k}}$ for $\mathrm{OAM}_{-2}$. Four gradients are multiplied by the weights $\alpha_{n}$ and finally summed up to get the total gradient. $\alpha_{n}$ is utilized to adjust the intensity disparities arising from the light field distribution of distinct modes and to make up for the modes featuring lower transmittance [5-7].

$$\begin{aligned} \alpha_{n}=\frac{\frac{\partial f_{n}}{\partial\varepsilon^{k}}}{\min\left( \frac{\partial f_{n}}{\partial{\varepsilon_{i}}^{k}} \right)}\times\left( \sum_{m=1}^{N} T_{m}-T_{n} \right)\#\left( S15 \right) \end{aligned}$$

First the greyscale phase where the parameters vary continuously between the index of Si and SiO_2_. In this process the figure of merit, which is related to the conversion efficiency, is enhanced rapidly. The second step is partial-binarization optimization and the refractive index of the material units in the design region is controlled according to the bias factor $\beta$ which increases gradually to achieve binarization in this step.

$$\begin{aligned} \varepsilon_{i}=\varepsilon_{min}+\frac{\tanh\left( \beta\eta\right)+\tanh\left( \beta\left( z_{i}-\eta\right) \right)}{\tanh\left( \beta\eta\right)+\tanh\left( \beta\left( 1-\eta\right) \right)}\left( \varepsilon_{max}-\varepsilon_{min} \right)\#\left( S16 \right) \end{aligned}$$

We delineate several binarization-sensitive regions where $\beta$ varies slowly from 1 to 20 and other regions where $\beta$ increases fast to 100. After the completion of binarizing most area, we then binarize these several delineated areas for additional number of iterations. In the end, $\varepsilon_{i}$ becomes either the value of the refractive index of Si or SiO_2_. Before each update of the refractive index parameters, we utilize computer graphics transformations to eliminate islands and thin connecting parts smaller than a tunable threshold size, and fill holes below the same threshold. The threshold is determined by the minimum feature size constraint and in our design the minimum feature size of manufacturing tolerance is 200 nm.

A small footprint of the device size is preferred so the greyscale phase of several optimization regions with different sizes , from 0.53 μm×1.8 μm to 5 μm × 4 μm, have been tested. For the former small regions the optimization processes prove quite challenging and at the size of 5 μm × 4 μm, the first 20–30 iterations in grayscale phase perform much better with the FOM approaches approximately 40%. Finally we adopt this size is adopted to proceed with the further optimization.

In summary, to balance conversion efficiency, equipment size, and manufacturing tolerance in the inverse design of the OAM mode converters, we adopt optimization process refinement, enforce a 200 nm minimum feature size for manufacturing and gradually scale up the optimization region and ultimately achieve a well-balanced performance with significant FOM improvement and other metrics.

**S2.** **The curve fit for the** $\mathbf{OAM}_{\boldsymbol{m\leftrightarrow n}}$ **converter and multiplexer**

Considering the periodicity of electromagnetic waves during propagation, we employed sine and cosine functions to fit the conversion coefficients. In the following formulas, $\alpha_{mn}$ denotes the conversion efficiency from $\mathrm{OAM}_{l=m}$ to $\mathrm{OAM}_{l=n}$ ($m, n=1, -1, 2, -2$).

For $\mathrm{OAM}_{1\leftrightarrow2}$ Converter, the conversion curves are shown in figure 2(d-k). The conversion efficiency is expressed as:

$$\begin{aligned} \alpha_{12}=\sum_{n=0}^{3} A_{n}\sin\left( \frac{n\pi d}{d_{0}} \right)+B_{n}\cos\left( \frac{n\pi d}{d_{0}} \right)\#\left( S17 \right) \end{aligned}$$

$$\begin{aligned} \alpha_{-1-2}=\sum_{n=0}^{3} C_{n}\sin\left( \frac{n\pi d}{d_{0}} \right)+D_{n}\cos\left( \frac{n\pi d}{d_{0}} \right)\#\left( S18 \right) \end{aligned}$$

$$\begin{aligned} \alpha_{21}=\sum_{n=0}^{4} E_{n}\sin\left( \frac{n\pi d}{d_{0}} \right)+F_{n}\cos\left( \frac{n\pi d}{d_{0}} \right)\#\left( S18 \right) \end{aligned}$$

$$\begin{aligned} \alpha_{-2-1}=\sum_{n=0}^{4} G_{n}\sin\left( \frac{n\pi d}{d_{0}} \right)+H_{n}\cos\left( \frac{n\pi d}{d_{0}} \right)\#\left( S19 \right) \end{aligned}$$

$$\begin{aligned} A_{n}+B_{n}=C_{n}+D_{n}=E_{n}+F_{n}=G_{n}+H_{n}=1\#\left( S20 \right) \end{aligned}$$

The length of one variation period is $2d_{0}$

**Table S1. Coefficients of curve fit for the multiplexer** $\mathbf{OAM}_{\boldsymbol{1\leftrightarrow2}}$ **Converter**

| $\boldsymbol{n}$ | $\mathbf{0}$ | $\mathbf{1}$ | $\mathbf{2}$ | $\mathbf{3}$ | $\mathbf{4}$ | $\boldsymbol{R}^{\mathbf{2}}$ | $\mathbf{2}\boldsymbol{d}_{\mathbf{0}}\mathbf{(}\boldsymbol{\mu m}\mathbf{)}$ |
| --- | --- | --- | --- | --- | --- | --- | --- |
| $A_{n}$ | 0.31273 | -0.057405 | 0.014986 | -0.009868 | 0 | 0.9335 | 8.60 |
| $C_{n}$ | 0.32691 | -0.068077 | 0.021168 | 0.0022522 | 0 | 0.9909 |  |
| $E_{n}$ | 0.60143 | 0.02233 | 0.044629 | 0.12928 | -0.011494 | 0.9581 | 2.32 |
| $G_{n}$ | 0.66547 | -0.039701 | -0.0099782 | 0.10113 | 0.0024999 | 0.9885 |  |

For $\mathrm{OAM}_{2\leftrightarrow3}$ Converter, the conversion curves $\mathrm{OAM}_{+2\to+3}$ and $\mathrm{OAM}_{-2\to-3}$ are shown in figure 3(e-h). The conversion curves $\mathrm{OAM}_{+3\to+2}$ and $\mathrm{OAM}_{-3\to-2}$ are shown in figure S1(a)(b).


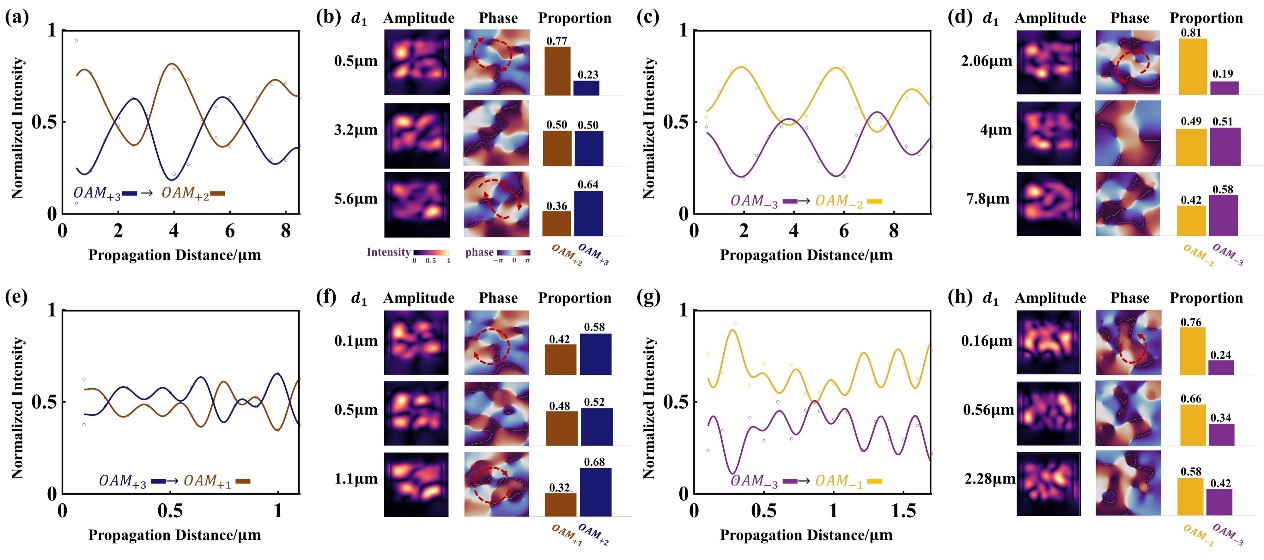


Figure S1. The conversion curves and the output light fields of OAM mode converters. (a) Periodic evolution of the mode fractions for $\mathrm{OAM}_{l=+3}$ into $\mathrm{OAM}_{l=+2}$ with the output distance. (c) Periodic evolution of the mode fractions for $\mathrm{OAM}_{l=-3}$ into $\mathrm{OAM}_{l=-2}$ with the output distance. (b)(d) is the intensity, phase, and proportion diagrams of the output light fields at three different positions.

The conversion efficiency is expressed as:

$$\begin{aligned} \alpha_{23}=\sum_{n=0}^{7} A_{n}\sin\left( \frac{n\pi d}{d_{0}} \right)+B_{n}\cos\left( \frac{n\pi d}{d_{0}} \right)\#\left( S17 \right) \end{aligned}$$

$$\begin{aligned} \alpha_{-2-3}=\sum_{n=0}^{6} C_{n}\sin\left( \frac{n\pi d}{d_{0}} \right)+D_{n}\cos\left( \frac{n\pi d}{d_{0}} \right)\#\left( S18 \right) \end{aligned}$$

$$\begin{aligned} \alpha_{32}=\sum_{n=0}^{6} E_{n}\sin\left( \frac{n\pi d}{d_{0}} \right)+F_{n}\cos\left( \frac{n\pi d}{d_{0}} \right)\#\left( S18 \right) \end{aligned}$$

$$\begin{aligned} \alpha_{-3-2}=\sum_{n=0}^{4} G_{n}\sin\left( \frac{n\pi d}{d_{0}} \right)+H_{n}\cos\left( \frac{n\pi d}{d_{0}} \right)\#\left( S19 \right) \end{aligned}$$

**Table S2. Coefficients of curve fit for the multiplexer** $\mathbf{OAM}_{\boldsymbol{2\leftrightarrow3}}$ **Converter**

| $\boldsymbol{n}$ | $\mathbf{0}$ | $\mathbf{1}$ | $\mathbf{2}$ | $\mathbf{3}$ | $\boldsymbol{R}^{\mathbf{2}}$ |
| --- | --- | --- | --- | --- | --- |
| $A_{n}$ | 0.5022 | -0.17413 | -0.06412 | -0.058169 | 0.8223 |
| $C_{n}$ | 0.48327 | -0.11233 | -0.04519 | -0.04471 | 0.7781 |
| $E_{n}$ | 0.59607 | 0.022681 | -0.026925 | 0.048982 | 0.8234 |
| $G_{n}$ | 0.62556 | 0.030123 | 0.08551 | -0.11051 | 0.9309 |

| $\boldsymbol{n}$ | $\mathbf{4}$ | $\mathbf{5}$ | **6** | **7** | $\mathbf{2}\boldsymbol{d}_{\mathbf{0}}\mathbf{(}\boldsymbol{\mu m}\mathbf{)}$ |
| --- | --- | --- | --- | --- | --- |
| $A_{n}$ | -0.007521 | 0.030327 | 0.027692 | -0.054369 | 20.09 |
| $C_{n}$ | -0.022003 | -0.034695 | 0.014287 | 0 | -0.022003 |
| $E_{n}$ | -0.013522 | 0.011993 | -0.001716 | 0 | 8.50 |
| $G_{n}$ | -0.02205 | 0 | 0 | 0 | 9.50 |

For $\mathrm{OAM}_{1\leftrightarrow3}$ Converter, the conversion curves $\mathrm{OAM}_{+1\to+3}$ and $\mathrm{OAM}_{-1\to-3}$ are shown in figure 3(i-l). The conversion curves $\mathrm{OAM}_{+3\to+1}$ and $\mathrm{OAM}_{-3\to-1}$ are shown in figure S1(c)(d).

The conversion efficiency is expressed as:

$$\begin{aligned} \alpha_{13}=\sum_{n=0}^{6} A_{n}\sin\left( \frac{n\pi d}{d_{0}} \right)+B_{n}\cos\left( \frac{n\pi d}{d_{0}} \right)\#\left( S17 \right) \end{aligned}$$

$$\begin{aligned} \alpha_{-1-3}=\sum_{n=0}^{3} C_{n}\sin\left( \frac{n\pi d}{d_{0}} \right)+D_{n}\cos\left( \frac{n\pi d}{d_{0}} \right)\#\left( S18 \right) \end{aligned}$$

$$\begin{aligned} \alpha_{31}=\sum_{n=0}^{7} E_{n}\sin\left( \frac{n\pi d}{d_{0}} \right)+F_{n}\cos\left( \frac{n\pi d}{d_{0}} \right)\#\left( S18 \right) \end{aligned}$$

$$\begin{aligned} \alpha_{-3-1}=\sum_{n=0}^{7} G_{n}\sin\left( \frac{n\pi d}{d_{0}} \right)+H_{n}\cos\left( \frac{n\pi d}{d_{0}} \right)\#\left( S19 \right) \end{aligned}$$

$$\begin{aligned} A_{n}+B_{n}=C_{n}+D_{n}=E_{n}+F_{n}=G_{n}+H_{n}=1\#\left( S20 \right) \end{aligned}$$

The length of one variation period is $2d_{0}$

**Table S3. Coefficients of curve fit for** $\mathbf{OAM}_{\boldsymbol{1\leftrightarrow3}}$ **Converter**

| $\boldsymbol{n}$ | $\mathbf{0}$ | $\mathbf{1}$ | $\mathbf{2}$ | $\mathbf{3}$ | $\boldsymbol{R}^{\mathbf{2}}$ |
| --- | --- | --- | --- | --- | --- |
| $A_{n}$ | 0.48883 | -0.01705 | -0.04371 | -0.042374 | 0.8785 |
| $C_{n}$ | 0.70686 | 0.011466 | 0.051298 | 0.026135 | 0.9771 |
| $E_{n}$ | 0.4899 | 0 | 0.043204 | 0 | 0.9081 |
| $G_{n}$ | 0.65865 | 0.015196 | 0.017527 | 0.012553 | 0.7505 |

| $\boldsymbol{n}$ | $\mathbf{4}$ | $\mathbf{5}$ | **6** | **7** | $\mathbf{2}\boldsymbol{d}_{\mathbf{0}}\mathbf{(}\boldsymbol{\mu m}\mathbf{)}$ |
| --- | --- | --- | --- | --- | --- |
| $A_{n}$ | 0.0011957 | -0.066772 | -0.027019 | 0 | 1.30 |
| $C_{n}$ | 0 | 0 | 0 | 0 | 2.20 |
| $E_{n}$ | 0.05659 | 0.0071525 | 0 | 0.015671 | 1.10 |
| $G_{n}$ | -0.037189 | 0.00095937 | 0.023887 | 0.024251 | 1.70 |

For the multiplexer for $\mathrm{OAM}_{\pm1}$ and $\mathrm{OAM}_{\pm2}$, the conversion curves are shown in figure S4.

The conversion efficiency is expressed as:

$$\begin{aligned} \beta_{1}=\sum_{n=0}^{2} A_{n}\sin\left( \frac{n\pi d}{d_{0}} \right)+B_{n}\cos\left( \frac{n\pi d}{d_{0}} \right)\#\left( S21 \right) \end{aligned}$$

$$\begin{aligned} \beta_{2}=\sum_{n=0}^{2} C_{n}\sin\left( \frac{n\pi d}{d_{0}} \right)+D_{n}\cos\left( \frac{n\pi d}{d_{0}} \right)\#\left( S21 \right) \end{aligned}$$

**Table S4. Coefficients of curve fit for the multiplexer**

| $\boldsymbol{n}$ | $\mathbf{0}$ | $\mathbf{1}$ | $\mathbf{2}$ | $\boldsymbol{R}^{\mathbf{2}}$ | $\mathbf{2}\boldsymbol{d}_{\mathbf{0}}\mathbf{(}\boldsymbol{\mu m}\mathbf{)}$ |
| --- | --- | --- | --- | --- | --- |
| $A_{n}$ | 0.49037 | 0.093826 | 0.0026459 | 0.9998 | 21.90 |
| $C_{n}$ | 0.4366 | -0.41508 | 0.051593 | 0.9958 | 9.4 |

**S3. Performance** **comparison among different neural network schemes**

We compare the hybrid OCNN, electronic convolutional neural network (ECNN), and electronic fully connected network (EFCN). Specifically, the EFCN used as a control directly maps the flattened input image data to the 10-category outputs. In contrast, both the hybrid OCNN and EFCN first pass through a convolutional layer with two convolution kernels, then flatten the convolved data and connect it to a fully connected layer to produce the output. For the identical MNIST and Fashion-MNIST datasets, with the same number of training epochs set, the performance comparisons of the final network models are as follows and the convergence of the hybrid OCNN, ECNN and EFCN for the two datasets are shown in figure 5.(b)(c).The final confusion matrix of the hybrid OCNN in these two datasets are shown in figure 5.(d)(e) The confusion matrix of the ECNN and EFCN are shown in figure S2.

**Table S5. Comparison of hybrid OCNN, ECNN, EFCN**

| **neural network** | | **Trainable parameter numbers** | **accuracy** | |
| --- | --- | --- | --- | --- |
|  |  |  | **MINST** | **Fashion-MINST** |
| EFCN | Batch size =128  Epoch=30 | 5760 | 93% | 84% |
| ECNN |  | 2888 | 95% | 93% |
| hybrid OCNN |  | 2884 | 98% | 86% |


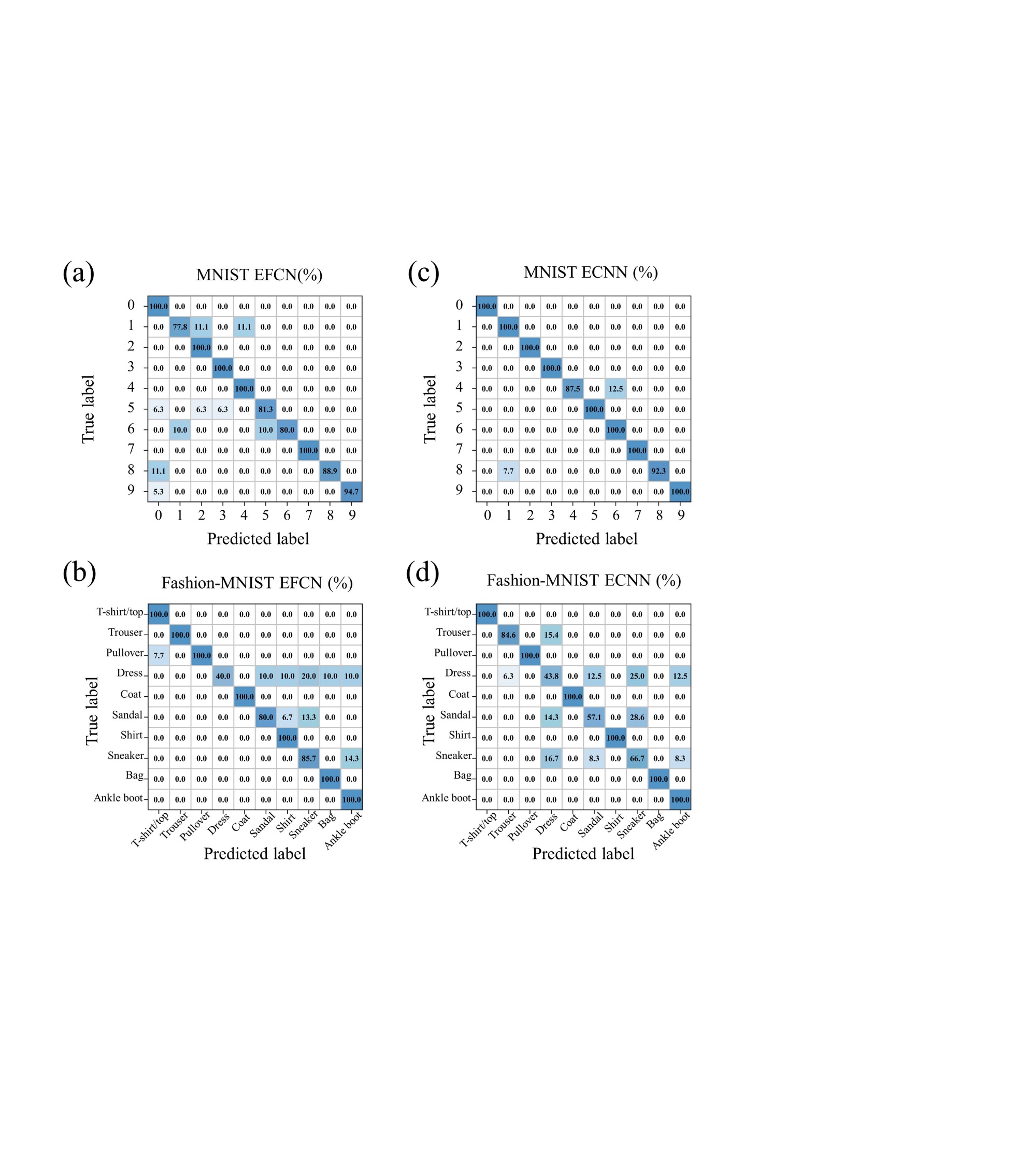


Figure S2. The confusion matrix of EFCN and ECNN. (a) The confusion matrix of EFCN in MNIST handwritten digit dataset. (b) The confusion matrix of the EFCN in Fashion-MNIST clothing item dataset. (c)The confusion matrix of the ECNN in MNIST handwritten digit dataset. (d) The confusion matrix of the ECNN in Fashion-MNIST clothing item dataset.

From the comparative results, convolutional networks significantly reduce the number of parameters during training compared to fully connected networks, thereby lowering the consumption of computational resources. It can be observed that compared with the EFCN, both the hybrid OCNN and the ECNN exhibit superior efficiency in data feature extraction and enhanced parameter optimization capabilities in image processing applications, owing to the introduction of convolutional layers.

**S4. Feasibility Analysis of OAM Detection and Demultiplexing**

**Supplementary Materials S4. Feasibility Analysis of OAM Detection and Demultiplexing**

The scheme 1 is an all-optical experimental detection scheme with specially designed diffraction gratings [8,9]. The grating applies spiral phase modulation to the OAM modes and different OAM orders correspond to different diffraction angles which form a discrete spot array in the far field or focal plane with each spot corresponding to one OAM order. After spatial separation, detection is accomplished via a detector array or single-point scanning.

The scheme 2 is an optoelectronic-integrated scheme. The SLM is used to generate the normalized standard OAM mode to make projection measurement for the output modes [10,11]. The output of the laser source is spilt into two beams: one serves as the reference beam which goes through the delay line and the SLM, the other is incident on the input signal source of the OAM chip. The input light is focused by an optical objective lens before being incident on the waveguide facet of the chip, such that its spot size matches the micron-scale on-chip waveguides. Similarly, the output OAM light is restored to its original size by an optical lens and then it is combined with the reference beams by a beam splitter. If the order of the normalized standard OAM mode is opposite to the output state, the result of the projection is the Gaussian component with planar phase front. To select this Gaussian component, we couple the light into a single-mode fiber connected to a power meter to measure the OAM power after the projection. And the measured power is normalized to get the final power spectrum of the output modes.

For example, the output filed is

$$\begin{aligned} f_{output}=a_{+m}E_{OAM}\left( A,\varphi,l=+m \right)+a_{-m}E_{OAM}\left( A,\varphi,l=-m \right)+ \\ a_{+n}E_{OAM}\left( A,\varphi,l=+n \right)+a_{-n}E_{OAM}\left( A,\varphi,l=-n \right)\#\left( S23 \right) \end{aligned}$$

and the reference fields is

$$\begin{aligned} f_{k}=E_{OAM}\left( A,\varphi,l=k \right)\#\left( S24 \right) \end{aligned}$$

If $k=+m$,the projection field is

$$\begin{aligned} f_{projection}=a_{+m}E_{OAM}\left( A,\varphi,l=0 \right)+a_{-m}E_{OAM}\left( A,\varphi,l=-2m \right)+ \\ a_{+n}E_{OAM}\left( A,\varphi,l=-m+n \right)+a_{-n}E_{OAM}\left( A,\varphi,l=-m-n \right)\#\left( S25 \right) \end{aligned}$$

$a_{+m}E\left( A,\varphi,l=0 \right)$ is the Gaussian component which can be coupled into a single-mode fiber and then $\left| a_{+m} \right|^{2}$ is measured by a power meter. We successively set the topological charge $k$ of the reference light to $-m$,$+n$, and $-n$ using SLM, thereby obtaining $a_{+m}$, $a_{-m}$,$a_{+n}$, and $a_{-n}$. From this, the content of each OAM order in the output light field can be determined.

**S5. Power spectrums of three converters and themultiplexe**r

The output field is expressed as:

$$\begin{aligned} f_{out}=\sum_{l} a_{l}E_{OAM}\left( r,\varphi,l \right)\#\left( S26 \right) \end{aligned}$$

where $E_{n}\left( r,\varphi,l \right)$ denotes the electric field of the normalized standard OAM mode and $a_{l}$ is the weight coefficient that can be calculated by overlap integral:

$$\begin{aligned} a_{l}=\iint{E_{n}\left( r,\varphi,l \right)}^{*}\cdot f_{out}\left( r,\phi,z \right)ds\#\left( S27 \right) \end{aligned}$$

${{|a}_{l}|}^{2}$  represents the power fraction of the$l$ th order OAM mode in the output optical field. The mode purity of the converter $\mathrm{OAM}_{m\to n}$ is defined as the power proportion of $\mathrm{OAM}_{n}$ at the output port of the converter $\mathrm{OAM}_{m\to n}$

$$\begin{aligned} \mathrm{Purity}_{m\to n}=\frac{{a_{l=n}}^{2}}{\sum_{i=-\infty}^{+\infty} {a_{l=i}}^{2}}\#\left( S28 \right) \end{aligned}$$

The crosstalk is defined as the other modes at the output port.

$$\begin{aligned} crosstalk=10\lg\left( 1-\frac{{a_{l=n}}^{2}}{\sum_{i=-\infty}^{+\infty} {a_{l=i}}^{2}} \right)\#\left( S29 \right) \end{aligned}$$

The power spectrums${{|a}_{l}|}^{2}$ of three converters $\mathrm{OAM}_{1\leftrightarrow2}$ Converter,$\mathrm{OAM}_{2\leftrightarrow3}$ Converter,$\mathrm{OAM}_{1\leftrightarrow3}$ Converter and the $\mathrm{OAM}_{1,2}$ multiplexer are in Figure S3(a-d). It shows that in terms of the absolute content of each order, the target conversion orders have relatively high content.


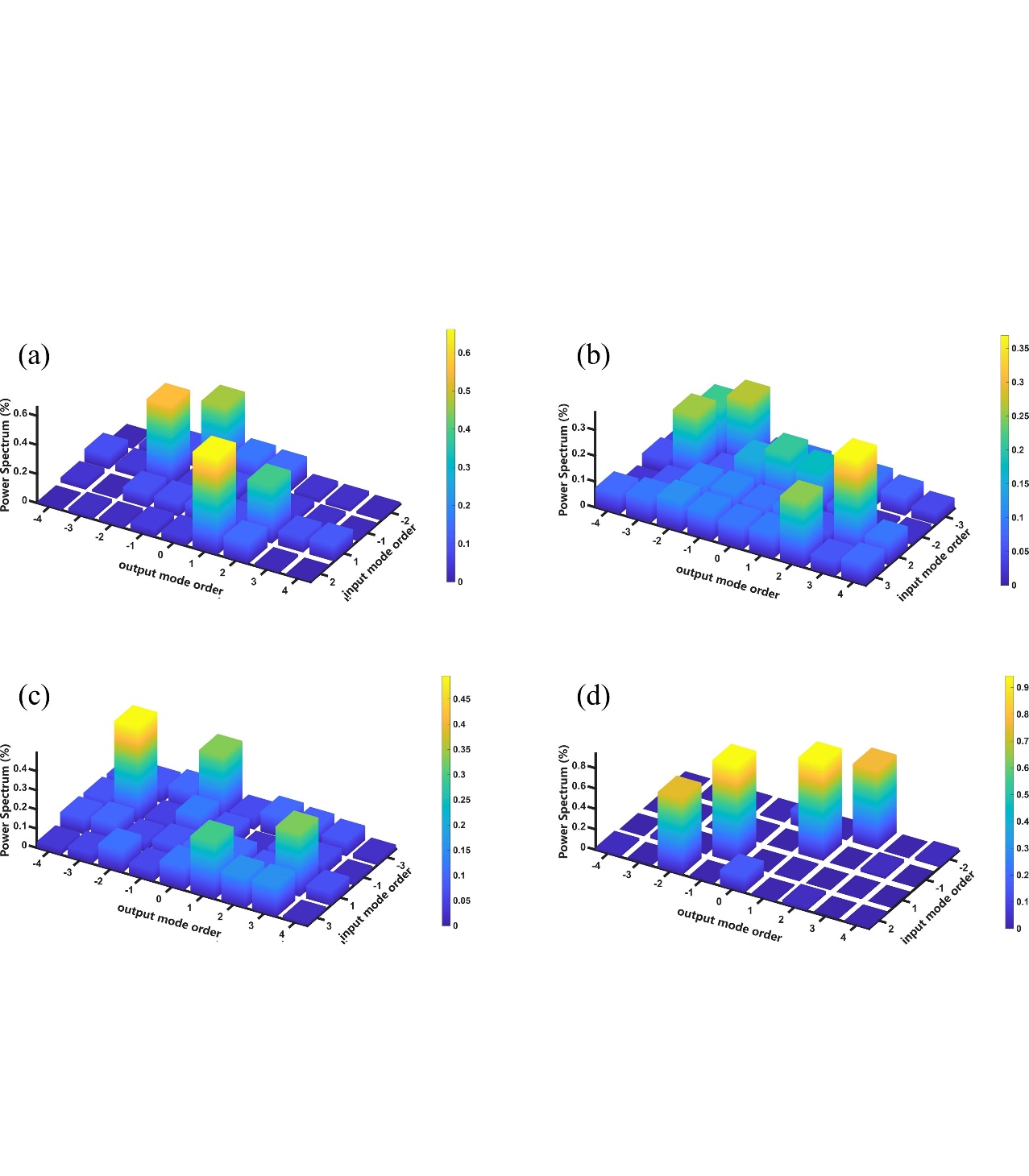


Figure S3. Power spectrums of (a)$\mathrm{OAM}_{1\leftrightarrow2}$ Converter, (b)$\mathrm{OAM}_{2\leftrightarrow3}$ Converter, (c)$\mathrm{OAM}_{1\leftrightarrow3}$ Converter and (d)the $\mathrm{OAM}_{1,2}$ multiplexer.

Purity and Crosstalk of three $\mathrm{OAM}_{m\to n}$ converters and the $\mathrm{OAM}_{m,n}$ multiplexer are shown in Table S6.

Table S6. Purity and Crosstalk of the converter $\mathrm{OAM}_{m\to n}$ and the $\mathrm{OAM}_{m,n}$ multiplexer

| Converter | | Up-conversion | | Down-conversion | |
| --- | --- | --- | --- | --- | --- |
|  |  | Purity | Crosstalk | Purity | Crosstalk |
| $\mathrm{OAM}_{1\leftrightarrow2}$ | $+1\leftrightarrow+2$ | $66.15\%$ | -4.7044 dB | $39.13\%$ | -2.1560 dB |
|  | $-1\leftrightarrow-2$ | $55.72\%$ | -3.5379 dB | $46.56\%$ | -2.7213 dB |
| $\mathrm{OAM}_{2\leftrightarrow3}$ | $+2\leftrightarrow+3$ | $36.91\%$ | -2.0004 dB | $25.08\%$ | -1.2540 dB |
|  | $-2\leftrightarrow-3$ | $25.79\%$ | -1.2954 dB | $26.69\%$ | -1.3484 dB |
| $\mathrm{OAM}_{1\leftrightarrow3}$ | $+1\leftrightarrow+3$ | $32.91\%$ | -1.7334 dB | $29.39\%$ | -1.5113 dB |
|  | $-1\leftrightarrow-3$ | $49.59\%$ | -2.9748 dB | $33.28\%$ | -1.7574 dB |
| $\mathrm{OAM}_{1,2}$ multiplexer | $-1\leftrightarrow+1$ | $94.33\%$ | -12.4642 dB | $94.34\%$ | -12.4718 dB |
|  | $-2\leftrightarrow+2$ | $76.94\%$ | -6.3714 dB | $74.40\%$ | -5.9176 dB |

**S6. Robustness Analysis Under Crosstalk**

S6.1 Crosstalk resistance analysis

In this part, crosstalk has been incorporated into the training results of the neural network and accordingly the structure of the pre-trained network and the parameters in the weight matrix remain unchanged while the crosstalk term has been added to the pre-trained weight matrix of the hybrid OCNN neural network.

$$w_{1-ct}=w_{1}+w_{ct}=\left[ \begin{matrix} \begin{matrix} p_{+m\to+n}\left( 1-\alpha_{mn} \right) & 0 \\ 0 & p_{-m\to-n}\left( 1-\alpha_{-m-n} \right) \end{matrix} & \begin{matrix} p_{+m\to+n}\alpha_{mn} & 0 \\ 0 & p_{-m\to-n}\alpha_{-m-n} \end{matrix} \\ \begin{matrix} p_{+n\to+m}\alpha_{nm} & 0 \\ 0 & p_{-n\to-m}\alpha_{-n-m} \end{matrix} & \begin{matrix} p_{+n\to+m}\left( 1-\alpha_{nm} \right) & 0 \\ 0 & p_{-n\to-m}\left( 1-\alpha_{-n-m} \right) \end{matrix} \end{matrix} \right]+\left[ \begin{matrix} \begin{matrix} 0 & \mathrm{ct}_{-m,+m\to+n} \\ \mathrm{ct}_{+m,-m\to-n} & 0 \end{matrix} & \begin{matrix} 0 & \mathrm{ct}_{-n,+m\to+n} \\ \mathrm{ct}_{+n,-m\to-n} & 0 \end{matrix} \\ \begin{matrix} 0 & \mathrm{ct}_{+m,+n\to+m} \\ \mathrm{ct}_{-m,-n\to-m} & 0 \end{matrix} & \begin{matrix} 0 & \mathrm{ct}_{+n,+n\to+m} \\ \mathrm{ct}_{-n,-n\to-m} & 0 \end{matrix} \end{matrix} \right]$$

$$\begin{aligned} =\left[ \begin{matrix} \begin{matrix} p_{+m\to+n}\left( 1-\alpha_{mn} \right) & \mathrm{ct}_{-m,+m\to+n} \\ \mathrm{ct}_{+m,-m\to-n} & p_{-m\to-n}\left( 1-\alpha_{-m-n} \right) \end{matrix} & \begin{matrix} p_{+m\to+n}\alpha_{mn} & \mathrm{ct}_{-n,+m\to+n} \\ \mathrm{ct}_{+n,-m\to-n} & p_{-m\to-n}\alpha_{-m-n} \end{matrix} \\ \begin{matrix} p_{+n\to+m}\alpha_{nm} & \mathrm{ct}_{+m,+n\to+m} \\ \mathrm{ct}_{-m,-n\to-1} & p_{-n\to-m}\alpha_{-n-m} \end{matrix} & \begin{matrix} p_{+n\to+m}\left( 1-\alpha_{nm} \right) & \mathrm{ct}_{+n,+n\to+m} \\ \mathrm{ct}_{-n,-n\to-m} & p_{-n\to-m}\left( 1-\alpha_{-n-m} \right) \end{matrix} \end{matrix} \right]\#\left( S30 \right) \end{aligned}$$

$$w_{2-ct}=w_{2}+w_{ct}=\left[ \begin{matrix} \begin{matrix} p_{+m\to-m}\beta_{1} & p_{+m\to-m}\left( 1-\beta_{1} \right) \\ p_{-m\to+m}\left( 1-\beta_{1} \right) & p_{-m\to+m}\beta_{1} \end{matrix} & \begin{matrix} 0 & 0 \\ 0 & 0 \end{matrix} \\ \begin{matrix} 0 & 0 \\ 0 & 0 \end{matrix} & \begin{matrix} p_{+n\to-n}\beta_{2} & p_{+n\to-n}\left( 1-\beta_{2} \right) \\ p_{-n\to+n}\left( 1-\beta_{2} \right) & p_{-n\to+n}\beta_{2} \end{matrix} \end{matrix} \right]+\left[ \begin{matrix} \begin{matrix} 0 & 0 \\ 0 & 0 \end{matrix} & \begin{matrix} \mathrm{ct}_{+n,+m\to-m} & \mathrm{ct}_{-n,+m\to-m} \\ \mathrm{ct}_{+n,-m\to+m} & \mathrm{ct}_{-n,-m\to+m} \end{matrix} \\ \begin{matrix} \mathrm{ct}_{+m,+n\to-n} & \mathrm{ct}_{-m,+n\to-n} \\ \mathrm{ct}_{+m,-n\to+n} & \mathrm{ct}_{-m,-n\to+n} \end{matrix} & \begin{matrix} 0 & 0 \\ 0 & 0 \end{matrix} \end{matrix} \right]$$

$$\begin{aligned} =\left[ \begin{matrix} \begin{matrix} p_{+m\to-m}\beta_{1} & p_{+m\to-m}\left( 1-\beta_{1} \right) \\ p_{-m\to+m}\left( 1-\beta_{1} \right) & p_{-m\to+m}\beta_{1} \end{matrix} & \begin{matrix} \mathrm{ct}_{+n,+m\to-m} & \mathrm{ct}_{-n,+m\to-m} \\ \mathrm{ct}_{+n,-m\to+m} & \mathrm{ct}_{-n,-m\to+m} \end{matrix} \\ \begin{matrix} \mathrm{ct}_{+m,+n\to-n} & \mathrm{ct}_{-m,+n\to-n} \\ \mathrm{ct}_{+m,-n\to+n} & \mathrm{ct}_{-m,-n\to+n} \end{matrix} & \begin{matrix} p_{+n\to-n}\beta_{2} & p_{+n\to-n}\left( 1-\beta_{2} \right) \\ p_{-n\to+n}\left( 1-\beta_{2} \right) & p_{-n\to+n}\beta_{2} \end{matrix} \end{matrix} \right]\#\left( S31 \right) \end{aligned}$$

$w_{1-ct}$,$w_{2-ct}$ are the transmission matrixes considering crosstalk of the on-chip converter $\mathrm{OAM}_{m\leftrightarrow n}$ and the $\mathrm{OAM}_{m,n}$ multiplexer. $w_{1}$,$w_{2}$ are the transmission matrixes under ideal conditions without crosstalk.$p_{i\to j}$ denotes the sum of the power proportions of $\mathrm{OAM}_{i}$ and $\mathrm{OAM}_{j}$ in the output light field when $\mathrm{OAM}_{i\to j}$  converter achieves the maximum conversion efficiency for $\mathrm{OAM}_{j}$. It represents the content of these two OAM modes actually involved in the computation.$\mathrm{ct}_{l,i\to j}$ refers to the maximum power proportion of the OAM mode with order $l$ in the output light field during the entire conversion process. The values of the purity and crosstalk parameters are presented in the following tables Table S7 and Table S8.

Table S7. $p_{i\to j}$ and $\mathrm{ct}_{l,i\to j}$ of the $\mathrm{OAM}_{1\leftrightarrow2}$  converter

|  | $i=+1,$  $j=+2$ | $i=-1,$  $j=-2$ | $i=+2,$  $j=+1$ | $i=-2,$  $j=-1$ |
| --- | --- | --- | --- | --- |
| $p_{i\to j}$ | 0.4770 | 0.6069 | 0.8033 | 0.5560 |
| $\mathrm{ct}_{l=-i,i\to j}$ | 0.0905 | 0.0643 | 0.0255 | 0.0371 |
| $\mathrm{ct}_{l=-j,i\to j}$ | 0.0940 | 0.0477 | 0.0533 | 0.1442 |

Table S8. $p_{i\to j}$ and $\mathrm{ct}_{l,i\to j}$ of the $\mathrm{OAM}_{1,2}$  multiplexer

|  | $i=+1$ | $i=-1$ | $i=+2$ | $i=-2$ |
| --- | --- | --- | --- | --- |
| $p_{i\to-i}$ | 0.9577 | 0.9577 | 0.7785 | 0.7813 |
| $\mathrm{ct}_{l=j,i\to-i}$ | 0.0005 | 0.0002 | 0.0110 | 0.0091 |
| $\mathrm{ct}_{l=-j,i\to-i}$ | 0.0002 | 0.0005 | 0.0085 | 0.0115 |

The prediction of the neural network is re-performed using the weight matrix that accounts for crosstalk and the resulting confusion matrix and accuracy are presented in figure S3 and Table S9. Thus, it can be concluded that when crosstalk is taken into account, the performance of the neural network remains unaffected, indicating that our proposed scheme exhibits strong robustness against crosstalk.


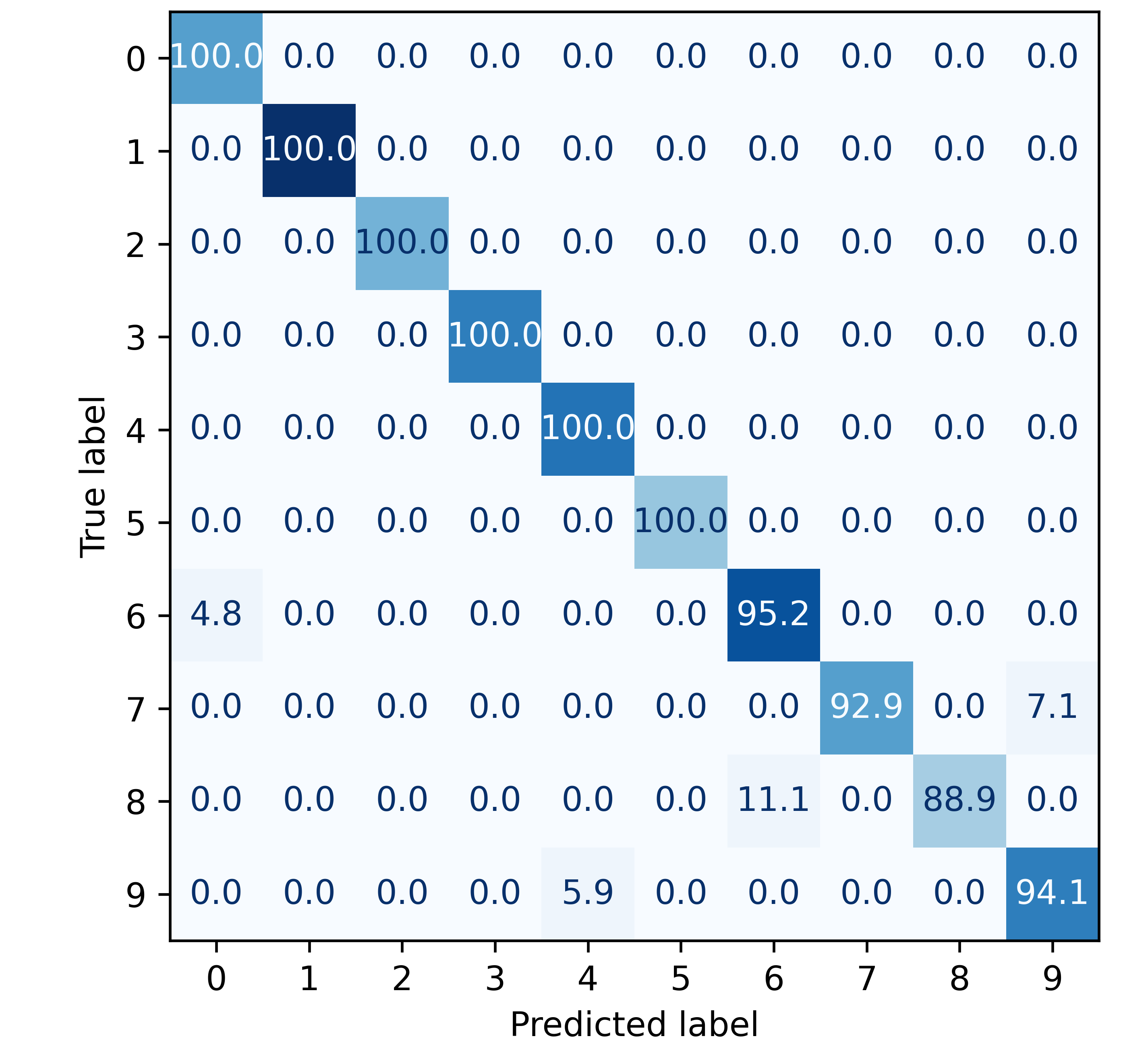

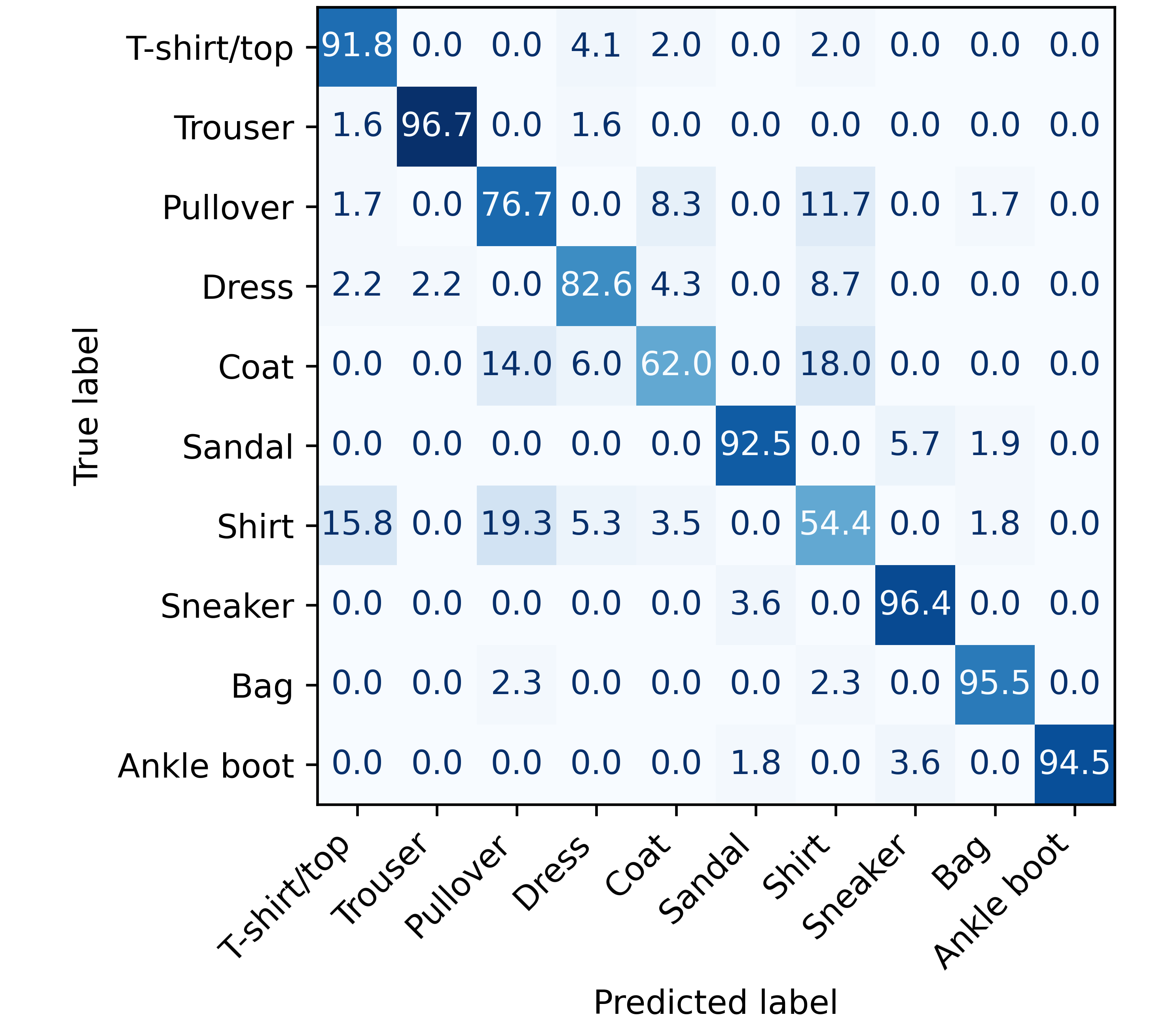


Fig S4. confusion matrix of the neural network with crosstalk. (a) MINST; (b) Fashion-MINST

Table S9. Accuracy of the neural network

|  | MINST | | Fashion-MINST | |
| --- | --- | --- | --- | --- |
|  | No Crosstalk | With Crosstalk | No Crosstalk | With Crosstalk |
| $\mathrm{accuracy}$ | $98\%$ | 97.3% | $86\%$ | 84.2% |

S6.2 Maximum crosstalk resistance

In this part the maximum crosstalk that can be tolerated by the hybrid OCNN is analyzed and another parameter, crosstalk coefficient $\rho$, is defined to theoretically derive the threshold of crosstalk.

The purity values presented in Table S7 and Table S8 are used while the crosstalk in the system is redefined as the product of purity and crosstalk coefficient $\rho\times purity$. The reconstructed crosstalk matrix and weight matrixes are as follows:

$$w_{1-ct}=w_{1}+w_{ct}=\left[ \begin{matrix} \begin{matrix} p_{+m\to+n}\left( 1-\alpha_{mn} \right) & 0 \\ 0 & p_{-m\to-n}\left( 1-\alpha_{-m-n} \right) \end{matrix} & \begin{matrix} p_{+m\to+n}\alpha_{mn} & 0 \\ 0 & p_{-m\to-n}\alpha_{-m-n} \end{matrix} \\ \begin{matrix} p_{+n\to+m}\alpha_{nm} & 0 \\ 0 & p_{-n\to-m}\alpha_{-n-m} \end{matrix} & \begin{matrix} p_{+n\to+m}\left( 1-\alpha_{nm} \right) & 0 \\ 0 & p_{-n\to-m}\left( 1-\alpha_{-n-m} \right) \end{matrix} \end{matrix} \right]+\left[ \begin{matrix} \begin{matrix} 0 & \rho p_{+m\to+n} \\ \rho p_{-m\to-n} & 0 \end{matrix} & \begin{matrix} 0 & \rho p_{+m\to+n} \\ \rho p_{-m\to-n} & 0 \end{matrix} \\ \begin{matrix} 0 & \rho p_{+n\to+m} \\ \rho p_{+n\to+m} & 0 \end{matrix} & \begin{matrix} 0 & \rho p_{+n\to+m} \\ \rho p_{+n\to+m} & 0 \end{matrix} \end{matrix} \right]$$

$$\begin{aligned} =\left[ \begin{matrix} \begin{matrix} p_{+m\to+n}\left( 1-\alpha_{mn} \right) & \rho p_{+m\to+n} \\ \rho p_{-m\to-n} & p_{-m\to-n}\left( 1-\alpha_{-m-n} \right) \end{matrix} & \begin{matrix} p_{+m\to+n}\alpha_{mn} & \rho p_{+m\to+n} \\ \rho p_{-m\to-n} & p_{-m\to-n}\alpha_{-m-n} \end{matrix} \\ \begin{matrix} p_{+n\to+m}\alpha_{nm} & \rho p_{+n\to+m} \\ \rho p_{+n\to+m} & p_{-n\to-m}\alpha_{-n-m} \end{matrix} & \begin{matrix} p_{+n\to+m}\left( 1-\alpha_{nm} \right) & \rho p_{+n\to+m} \\ \rho p_{+n\to+m} & p_{-n\to-m}\left( 1-\alpha_{-n-m} \right) \end{matrix} \end{matrix} \right]\#\left( S31 \right) \end{aligned}$$

$$w_{2-ct}=w_{2}+w_{ct}$$

$$\begin{aligned} =\left[ \begin{matrix} \begin{matrix} p_{+m\to-m}\beta_{1} & p_{+m\to-m}\left( 1-\beta_{1} \right) \\ p_{-m\to+m}\left( 1-\beta_{1} \right) & p_{-m\to+m}\beta_{1} \end{matrix} & \begin{matrix} \rho p_{+m\to-m} & \rho p_{+m\to-m} \\ \rho p_{-m\to+m} & \rho p_{-m\to+m} \end{matrix} \\ \begin{matrix} \rho p_{+n\to-n} & \rho p_{+n\to-n} \\ \rho p_{-n\to+n} & \rho p_{-n\to+n} \end{matrix} & \begin{matrix} p_{+n\to-n}\beta_{2} & p_{+n\to-n}\left( 1-\beta_{2} \right) \\ p_{-n\to+n}\left( 1-\beta_{2} \right) & p_{-n\to+n}\beta_{2} \end{matrix} \end{matrix} \right]\#\left( S32 \right) \end{aligned}$$

The curve of the network accuracy as crosstalk increases is shown in Figure S5. As can be seen from the curve, the accuracy shows a slight decrease with the increase of crosstalk. However, when the crosstalk coefficient is less than 0.4, the accuracy does not decrease significantly, indicating good robustness of the proposed scheme. In contrast, when the crosstalk is excessively large, the accuracy drops sharply.


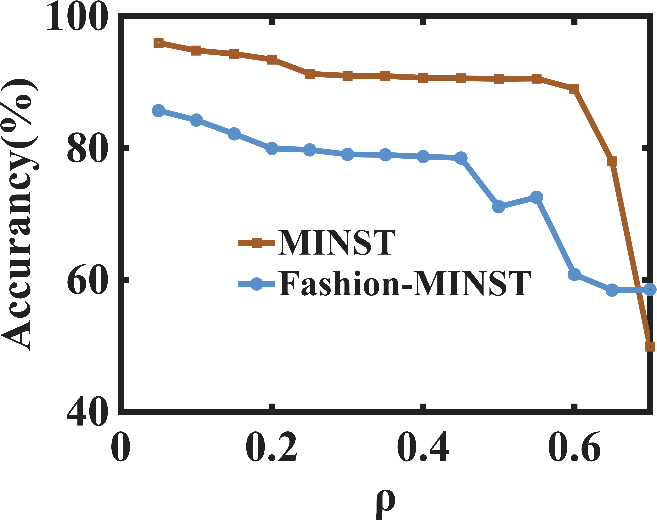


Figure S5. The variation curve of network performance as crosstalk increases

**S7. theoretical analysis of on-chip OAM order generation**

For an on-chip Laguerre-Gaussian OAM mode with topological charge $l$, the required Hermite-Gaussian mode components are determined by the relationship

$$\begin{aligned} e^{il\varphi}r^{l}L_{\rho}^{l}\left( r^{2} \right)=\frac{\left( -1 \right)^{\rho}}{2^{2\rho+l}l!}\sum_{m=0}^{\rho} \sum_{n=0}^{l} i^{n}\left( \begin{aligned} \rho\\ m \end{aligned} \right)\left( \begin{aligned} l \\ n \end{aligned} \right)H_{2m+l-n}\left( y \right)H_{2\rho-2m+n}\left( z \right)\#\left( S33 \right) \end{aligned}$$

This implies:


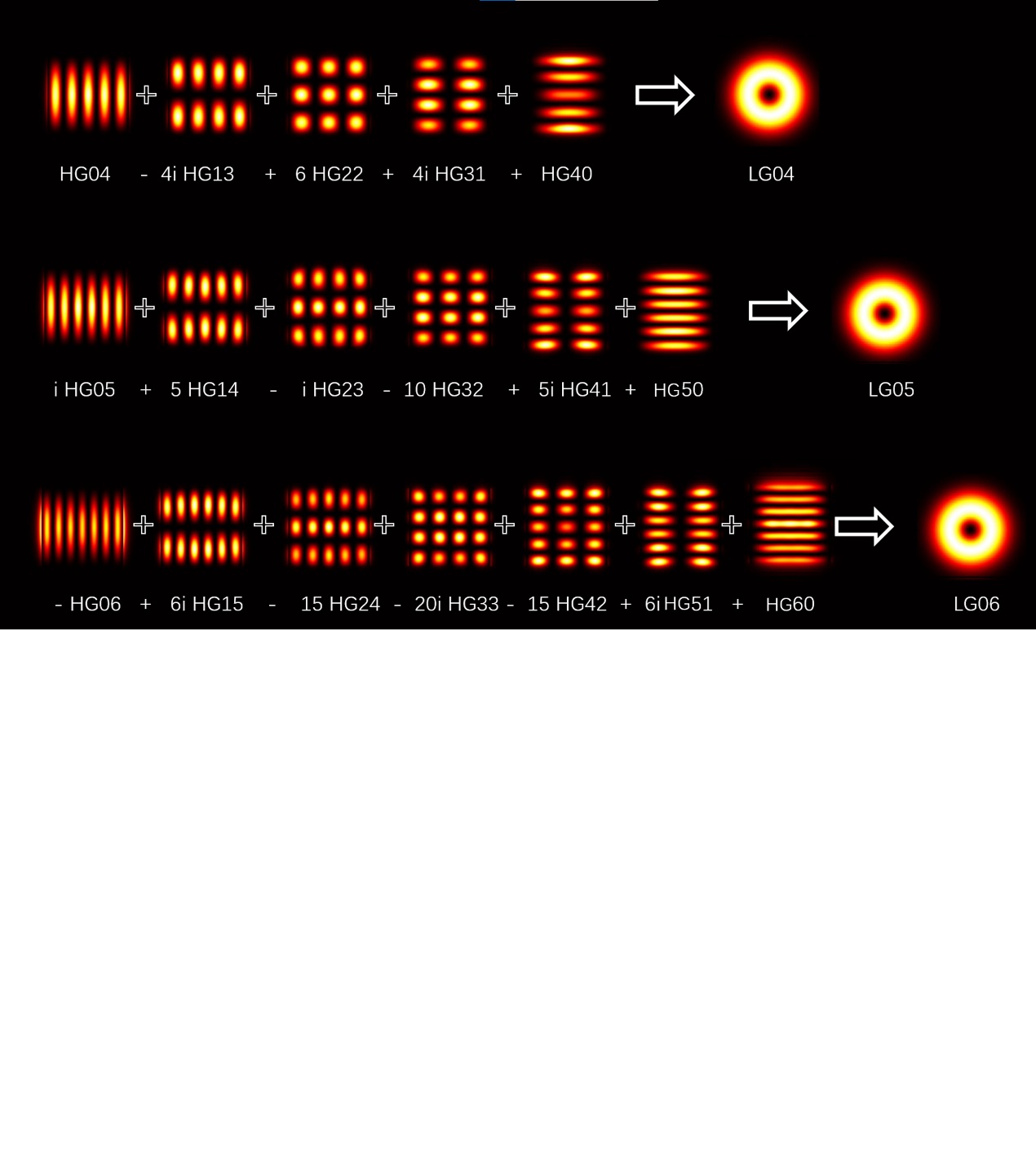


Figure S6. combination of HG_MN_ to generate OAM modes

$OAM l=0$ requires HG_00_

$OAM l=1$ requires HG_10_ HG_01_

$OAM l=2$ requires HG_20_ HG_11_ HG_02_

$OAM l=3$ requires HG_30_ HG_21_ HG_12_ HG_03_

$OAM l=4$ requires HG_40_ HG_31_ HG_22_ HG_13_ HG_04_

$OAM l=5$ requires HG_50_ HG_41_ HG_32_ HG_23_ HG_14_ HG_05_

$OAM l=6$ requires HG_60_ HG_51_ HG_42_ HG_33_ HG_24_ HG_15_ HG_06_

Firstly, for the Si waveguide with given width and thickness, the higher-order modes in the figure above are not supported so theoretical maximum OAM order depends on the size of the Si waveguide. The commonly use thickness for mature CMOS fabrication includes 220 nm, 340 nm. (The supported modes in these waveguides with different widths are shown in Table S10. Supported modes in waveguides). For the waveguide of 220 nm thick and 0.5$\mu m$ width in which HG_10_ HG_01_ are not supported, the only OAM mode are $OAM l=0$. For the waveguide of 340 nm thick and 0.5$\mu m$ width in which HG_01_ is not supported, the only OAM mode are $OAM l=0$. For the waveguide of 1.2$\mu m$ thick and 1.8$\mu m$ width in which HG_50_ are not supported so the OAM modes$l>4$ are not supported.

Table S10. Supported modes in waveguides

| Waveguide thickness | Waveguide width | Supported HG modes | OAM modes can be converted |
| --- | --- | --- | --- |
| 0.22$\mu m$ | 0.5$\mu m$ | HG_00_ | LG_00_ |
|  | 1$\mu m$ | HG_00_ HG_01_ | LG_00_ |
|  | 1.8$\mu m$ | HG_00_ HG_01_ HG_02_ HG_03_ | LG_00_ |
| 0.34$\mu m$ | 0.5$\mu m$ | HG_00_ HG_10_ | LG_00_ |
|  | 1$\mu m$ | HG_00_ HG_01_ HG_10_ HG_02_ HG_11_ HG_12_ | LG_00_ LG_01_ |
|  | 1.8$\mu m$ | HG_00_ HG_01_ HG_10_ HG_02_ HG_11_ HG_03_ HG_12_ HG_13_ HG_03_ HG_04_ | LG_00_ LG_01_ |
| 1.2$\mu m$ | 0.5$\mu m$ | HG_00_ HG_01_ HG_10_ HG_20_ HG_30_HG_40_ | LG_00_ LG_01_ |
|  | 1$\mu m$ | HG_00_ HG_01_ HG_10_ HG_02_ HG_11_ HG_20_ HG_31_ | LG_00_ LG_01_ LG_02_ |
|  | 1.8$\mu m$ | HG_00_ HG_01_ HG_10_ HG_02_ HG_11_ HG_20_ HG_03_ HG_12_ HG_21_ HG_30_ HG_04_ HG_13_ HG_22_ HG_31_ HG_40_ HG_05_ HG_14_ HG_23_ HG_32_ HG_41_ HG_34_ | LG_00_ LG_01_ LG_02_ LG_03_ LG_04_ |

Table S11 presents a comparison of the state-of-the-art research works about the generation and manipulation of on-chip OAM.

Table S11. Comparison of on-chip OAM schemes

| Ref | Physical dimensions | Maximum topological charges | Maximum  purity | Function |
| --- | --- | --- | --- | --- |
| [13] | waveguide diameter: 8 µm | 1 | 60% | waveguide supporting OAM modes |
| [14] | waveguide diameter: 12 µm | 2 | 93% | locking of OAM modes |
| [15] | waveguide length of 10 mm | 2 | Not given | OAM modes conversion |
| [16] | waveguide cross-section of 4.5 μm × 4.5 μm | 1 | 97.1% | OAM modes generation |
| [17] | waveguide cross-section of 6.5 μm × 6.5 μm,  total length of 6mm | 3 | 80% | OAM modes generation |
| This work | waveguide cross-section of 1.8 μm × 1.2 μm,  converter longitudinal section of 5 µm × 4 µm | 3 | 98% | OAM modes generation and computation |

Secondly, the higher order mode in waveguide is more unstable than lower order modes so the higher order mode will have more spatially distributed lobes and cross-talk than lower-order modes. During propagation through the optimization region, slight phase and amplitude mismatches between lobes of higher order modes accumulate, leading to distorted OAM phase profiles. Besides, higher-order OAM modes are more susceptible to contamination by lower-order modes. In conclusion, the primary factor affecting the supportable OAM orders on-chip is the waveguide thickness, which is determined by the silicon wafer thickness of the standard CMOS fabrication process.

Although on-chip OAM are limited to achieve relatively high topological orders, on-chip systems have an advantage in modulation speed compared with free-space schemes. First, the essence of information capacity lies in "the number of bits transmitted per unit time," which must be evaluated by considering both the modulation rate of the modulator and the number of multiplexing channels. Single-channel modulation rates of on-chip modulators[18-21]—such as silicon-based Mach-Zehnder modulators and micro-ring modulators—can reach 100 Gbps, whereas the modulation rates of free-space modulators, mainly liquid crystal SLMs and MEMS-based SLMs, are generally less than several hundred Mbps[22-26]. Second, the response time of integrated optical chips reaches the micro-nanosecond level, enabling nanosecond-level signal switching. In contrast, the regulation of free-space optical fields requires a long interaction distance, which limits their rate. As a result, free-on-chip OAM solutions have the potential to meet the real-time requirements of high-speed data transmission. Third, the CMOS process compatibility of on-chip OAM manipulation allows the integration of multi-channel high-speed units and further enables parallel modulation.

In summary, on-chip OAM, on the one hand, expands the number of channels for on-chip optical field manipulation to a certain extent by utilizing multiple orthogonal OAM modes; on the other hand, it can effectively integrate with on-chip high-speed modulation units, thereby providing a promising solution for high-speed information processing and optical computing with high computing density.

**References**

[1] T. W. Hughes et al., “Adjoint Method and Inverse Design for Nonlinear Nanophotonic Devices,” ACS Photonics, vol. 5, no. 12, pp. 4781–4787, 2018.

[2] C. M. Lalau-Keraly et al., “Adjoint shape optimization applied to electromagnetic design,” Opt. Express, vol. 21, no. 18, pp. 21693–21693, 2013.

[3] J. S. Jensen et al., “Topology optimization for nano-photonics,” Laser Photon. Rev., vol. 5, no. 2, pp. 308–321, 2010.

[4] D. A. Tortorelli et al., “Design sensitivity analysis: Overview and review,” Inverse Problems in Engineering, vol. 1, no. 1, pp. 71–105, 1994.

[5] H. Liu et al., “Self-adjusting inverse design method for nanophotonic devices,” Opt. Express, vol. 30, no. 21, pp. 38832–38832, 2022.

[6] S. Yang et al., “Ultracompact programmable inverse-designed nanophotonic devices based on digital subwavelength structures,” Appl. Opt., vol. 62, no. 15, pp. 3926–3926, 2023.

[7] A. R. Klein et al., “Designing metasurface optical interfaces for solid-state qubits using many-body adjoint shape optimization,” Opt. Express, vol. 32, no. 22, pp. 38504–38504, 2024.

[8] T. Lei et al, “Massive individual orbital angular momentum channels for multiplexing enabled by Dammann gratings,” Light: Sci. Appl., vol. 4, no. 3, pp. e257, 2015.

[9] S. Fu et al, “Integrating 5 × 5 Dammann gratings to detect orbital angular momentum states of beams with the range of −24 to +24,” Appl. Opt., vol. 55, no. 7, pp. 1514–1517, 2016.

[10] J. Leach et al. “Measuring the orbital angular momentum of a single photon”, Phys. Rev. Lett., vol. 88, no. 25, pp. 257901, 2002.

[11] J. Leach et al. “Interferometric methods to measure orbital and spin, or the total angular momentum of a single photon”. Phys. Rev. Lett., vol. 92, no. 1, pp. 013601, 2004.

[12] I. J. Lee et al, “On-Chip Guiding of Higher-Order Orbital Angular Momentum Modes,” Photonics, vol. 6, no. 2, p. 72, 2019.

[13] Y. Chen et al., “Mapping Twisted Light into and out of a Photonic Chip,” Phys. Rev. Lett., vol. 121, no. 23, 2018

[14] N. Anonymous et al., “Locking Orbital Angular Momentum with Linear Momentum of Light,” Phys. Rev. Lett., vol. 135, no. 14, pp. 143802–143802, 2025.

[15] H. Qi et al., “Dynamically Encircling Exceptional Points in Different Riemann Sheets for Orbital Angular Momentum Topological Charge Conversion,” Phys. Rev. Lett., vol. 132, no. 24, pp. 243802–243802, 2024.

[16] J. Wang et al., “Tailoring light on three-dimensional photonic chips: a platform for versatile OAM mode optical interconnects,” Adv. Photon. vol. 5, no. 03, 2023.

[17] W. Zhao et al., “All-on-chip reconfigurable generation of scalar and vectorial orbital angular momentum beams,” Light: Sci. Appl., vol. 14, no. 1, pp. 227–227, 2025.

[18] C. Han et al., “Exploring 400 Gbps/λ and beyond with AI-accelerated silicon photonic slow-light technology,” Nature Commun., vol. 16, no. 1, pp. 6547–6547, 2025.

[19] C. Wang, Y. Cheng, Z. Xu, Q. Dai, and L. Fang, “Diffractive tensorized unit for million-TOPS general-purpose computing,” Nature Photon., vol. 19, no. 10, pp. 1078–1087, 2025.

[20] Z. Du et al., “Ultracompact and multifunctional integrated photonic platform,” Science Advances, vol. 10, no. 25, 2024.

[21] Wang, Y., Liao, K., Zhang, K. et al. Reconfigurable versatile integrated photonic computing chip. eLight vol.5, no. 20. 2025.

[22] C. Rosales-Guzmán et al. “Multiplexing 200 Spatial Modes with a Single Hologram.” J. Opt., vol. 19, no. 11, 5, pp. 113501–113501, 2017.

[23] S Zhou et al. “Intelligent Tailoring of a Broadband Orbital Angular Momentum Comb towards Efficient Optical Convolution.” Photon. Res., vol. 13, no. 5, p. 1148, 2025.

[24] X Fang et al. “Orbital Angular Momentum-Mediated Machine Learning for High-Accuracy Mode-Feature Encoding.” Light: Science & Applications, vol. 13, no. 1, 2024.

[25] Z Wan et al. “Divergence-Degenerate Spatial Multiplexing towards Future Ultrahigh Capacity, Low Error-Rate Optical Communications.” Light: Sci. Appl., vol. 11, no. 1, 2022.

[26] W Meng et al. “100 Hertz Frame-Rate Switching Three-Dimensional Orbital Angular Momentum Multiplexing Holography via Cross Convolution.” Opto-Electronic Science, vol. 1, no. 9, 2022.
